# Supplementary material for: Missed opportunities in hypertension risk factors screening in Indonesia: a mixed-methods evaluation of integrated health post (POSBINDU) implementation
Source: BMJ Open. 2022 Feb 21;12(2):e051315. doi: 10.1136/bmjopen-2021-051315 (PMC8862503; doi:10.1136/bmjopen-2021-051315)
Supplement: Author's manuscript [file bmjopen-2021-051315.draft_revisions.pdf]

BMJ Open

BMJ Open is committed to open peer review. As part of this commitment we make the peer review history of every article we publish publicly available.

When an article is published we post the peer reviewers' comments and the authors' responses online. We also post the versions of the paper that were used during peer review. These are the versions that the peer review comments apply to.

The versions of the paper that follow are the versions that were submitted during the peer review process. They are not the versions of record or the final published versions. They should not be cited or distributed as the published version of this manuscript.

BMJ Open is an open access journal and the full, final, typeset and author-corrected version of record of the manuscript is available on our site with no access controls, subscription charges or pay-per-view fees (http://bmjopen.bmj.com).

If you have any questions on BMJ Open's open peer review process please email info.bmjopen@bmj.com

BMJ Open

Missed Opportunities in Hypertension Screening in Indonesia: Evaluation of Integrated Screening Post Implementation

Journal:	*BMJ Open*
Manuscript ID	bmjopen-2021-051315
Article Type:	Original research
Date Submitted by the Author:	16-Mar-2021

Complete List of Authors:	Widyaningsih, Vitri; Universitas Sebelas Maret, Public Health<br>Mashuri, Yusuf; Universitas Sebelas Maret, Parasitology<br>Febrinasari, Ratih; Universitas Sebelas Maret, Pharmacology<br>Pamungkasari, Eti; Universitas Sebelas Maret, Public Health<br>Sumardiyono, Sumardiyono; Universitas Sebelas Maret, Public Health<br>Balgis, Balgis; Universitas Sebelas Maret, Public Health<br>Koot, Jaap; University Medical Centre Groningen, Department of Health Sciences<br>Landsman-Dijkstra, Jeanet; University Medical Centre Groningen, Department of Health Sciences<br>Probandari, Ari; Universitas Sebelas Maret,

Keywords:	Hypertension < CARDIOLOGY, International health services < HEALTH SERVICES ADMINISTRATION & MANAGEMENT, PUBLIC HEALTH, Quality in health care < HEALTH SERVICES ADMINISTRATION & MANAGEMENT

SCHOLARONE™
Manuscripts

BMJ

I, the Submitting Author has the right to grant and does grant on behalf of all authors of the Work (as defined in the below author licence), an exclusive licence and/or a non-exclusive licence for contributions from authors who are: i) UK Crown employees; ii) where BMJ has agreed a CC-BY licence shall apply, and/or iii) in accordance with the terms applicable for US Federal Government officers or employees acting as part of their official duties; on a worldwide, perpetual, irrevocable, royalty-free basis to BMJ Publishing Group Ltd ("BMJ") its licensees and where the relevant Journal is co-owned by BMJ to the co-owners of the Journal, to publish the Work in this journal and any other BMJ products and to exploit all rights, as set out in our licence.

The Submitting Author accepts and understands that any supply made under these terms is made by BMJ to the Submitting Author unless you are acting as an employee on behalf of your employer or a postgraduate student of an affiliated institution which is paying any applicable article publishing charge ("APC") for Open Access articles. Where the Submitting Author wishes to make the Work available on an Open Access basis (and intends to pay the relevant APC), the terms of reuse of such Open Access shall be governed by a Creative Commons licence – details of these licences and which Creative Commons licence will apply to this Work are set out in our licence referred to above.

Other than as permitted in any relevant BMJ Author's Self Archiving Policies, I confirm this Work has not been accepted for publication elsewhere, is not being considered for publication elsewhere and does not duplicate material already published. I confirm all authors consent to publication of this Work and authorise the granting of this licence.

For peer review only

Missed Opportunities in Hypertension Screening in Indonesia: Evaluation of Integrated Screening Post Implementation

Vitri Widyaningsih[1] , Yusuf Ari Mashuri[1], Ratih Puspita Febrinasari[1], Eti Poncorini Pamungkasari [1], Sumardiyono[1], Balgis[1], Jaap Koot[2], Jeanet Landsman-Dijkstra[2], Ari Probandari [1] on behalf of Scaling Up Non-Communicable Disease Intervention in South East Asia (SUNISEA) Project

[1]Faculty of Medicine, Universitas Sebelas Maret, Surakarta, Indonesia

[2] University of Groningen, University Medical Center Groningen, Department of Health Sciences, The Netherlands

Corresponding author: Prof. dr. Ari Natalia Probandari, MPH., Ph.D. Faculty of Medicine Universitas Sebelas Maret Surakarta, 57126, Central Java, Indonesia ari.probandari@staff.uns.ac.id
Phone: (0271) 646994

For peer review only

Abstract

Objectives: To assess the implementation and contextual barriers of POSBINDU in Indonesia.

Design: This was a concurrent mixed-methods study, with a cross-sectional analysis of secondary data and Focus Group Discussions on stakeholder of POSBINDU.

Setting: The study was conducted in seven districts in three provinces in Indonesia, with approximately 50% of the primary health care (PHC) were selected as areas for data collection (n PHC=100).

Participants: From 475 POSBINDU, we collected secondary data from 54,224 participants. For the qualitative approach, 21 focus group discussions (FGDs) and 2 in-depth interviews were held among a total of 223 informants.

Primary outcomes and measures: Proportion of POSBINDU visitors getting the hypertension screening and risk factors' assessment, and barriers of POSBINDU implementation.

Results: Out of the 114,581 POSBINDU visits from 54,224 participants, most (80%) were female and adults over 50 years old (50%) showing a suboptimal coverage on male and younger adults. Approximately 95.1% of visitors were measured for blood pressure in their first visit; 35.3% of whom had elevated blood pressure. Less than 25% of the visitors reported to be interviewed for risk factors during their first visit, less than 80% had anthropometric measurements, and less than 15% had blood cholesterol examinations. We revealed lack of resources and limited time to perform the complexities of activities and reporting as main barrier for effective hypertension screening in Indonesia.

Conclusions: This study showed missed opportunities in hypertension screening in Indonesia. The barriers include a lack of access and implementation barriers (capability, resources, and protocols).

Keywords Noncommunicable diseases, hypertension, screening, community-based

Article Summary

Findings

In a mixed-methods study, we found missed opportunities in hypertension screening in Indonesia. Several barriers include suboptimal coverage, complexities of activities and overlap between different NCD-related programs, and lack of resources.

Implications

For peer review only - http://bmjopen.bmj.com/site/about/guidelines.xhtml

There is a need to improve coverage and implementation of hypertension screening. An integrated approach to improve the implementation of hypertension screening, from guidelines to practice is crucial.

Strengths and Limitations of this study

- The strength of this study was the relatively large evaluation of POSBINDU using a mixed-methods study design. Hence, providing more comprehensive information on POSBINDU implementation.
- The study limitation includes the difficulty in differentiating whether the missed reporting was due to lack of activities or lack of reporting.
- Nevertheless, both the activities and reporting are important in NCD screening, particularly in the follow up.

Introduction

The increasing trends of Non-Communicable Diseases (NCDs) in the world, including Indonesia, require targeted and specific primary and secondary prevention.[1,2] Hypertension, one of the most common NCDs, has a relatively high (33.4%) prevalence in Indonesia.[3,4] These figures are estimated to increase even further with the changing (more sedentary) lifestyle, unhealthy diet, rising prevalence of obesity, and the increasing life expectancy.[5] In 2015, hypertension attributed to 41% of all disability-adjusted life-years (DALYs), and was the leading risk factor for cardiovascular diseases.[6] Economically, hypertension is accounted for $370 billion in medical costs per year worldwide.[7] Major modifiable risk factors for NCDs include smoking, alcohol consumption, unhealthy diet and obesity, and a sedentary lifestyle.[8,9] With the heavy burden and the economic cost of this disease, primary and secondary prevention for hypertension and its risk factors become very important.

In 2010, the WHO has recommended the implementation of Package of Essential Interventions for Non-Communicable (PEN) Diseases for low-middle income countries.[10] In response, the Ministry of Health (MOH) launched the Integrated Health Post (POSBINDU), as part of the PEN program in Indonesia. POSBINDU, a community-based program in hypertension screening and prevention[11], was added to the several existing NCD-related programs Indonesia. These include Prolanis (Program Pengendalian Penyakit Kronis), a community-based hypertension and diabetes management program affiliated with primary care[12] and Posyandu Lansia, a community-based NCD screening and management for the elderly.[13] Despite these efforts, the awareness and control of hypertension are still relatively low: only 25% of people with elevated blood pressure are aware of their condition, and only 54% of people diagnosed with hypertension take routine medication.[4,14,15] These conditions are still below the "rule of halves" for hypertension management, which recommends that 50% of hypertension patients be aware of their condition, with half of whom should be treated.[16,17]

A process evaluation is important in assessing the implementation, to identify barriers, and provide specific recommendations for improvement of POSBINDU. Previous studies have evaluated the effectiveness of the POSBINDU implementation.[11,18] However, they were lacking on the evaluation of contextual barriers in POSBINDU implementation. This study aims to portray the implementation of POSBINDU and its contextual barriers, to provide recommendations for better hypertension screening, and optimal linkage to care in Indonesia.

Methods

Setting

POSBINDU is a community-based activity run by community health cadres (volunteers) and supervised by primary health care (PHC) officials. POSBINDU aims to empower communities in screening for NCDs and the risk factors, targeting individuals above 15 years old, particularly those of productive

For peer review only - http://bmjopen.bmj.com/site/about/guidelines.xhtml

age.[19,20] The main activities include screening for NCDs (mainly hypertension and diabetes) and the risk factors (i.e., smoking, diet, physical activity, obesity). Further, POSBINDU also provides health education and facilitate referral to PHC.[19]

Study Design

This was a concurrent mixed-methods study in seven districts in three provinces in Indonesia (Central Java, East Java, and North Sumatra). We purposely selected provinces with relatively high prevalence of NCD based on a national health survey conducted in 2018.[21]

Ethnical approval

The study was approved by the ethical review board at Universitas Gadjah Mada (Ethical Clearance Number KE/FK/0648/EC/2019). The participants of Focus Group Discussion gave informed consent before participating in this study.

Data Collection

Within every one of the three provinces, we selected two districts: one city representing urban communities, and one district representing rural communities. In Central Java, an additional city was also selected. For each district, approximately 50% of the primary health care (PHC) were selected as areas for data collection (n PHC=100). Within the PHC, we collected data for quantitative process evaluation from all active POSBINDU in the areas (n POSBINDU=475). In most POSBINDU, online/electronic data were not available; Hence, data on participation were manually collected from the POSBINDU register. Data from 2018-2019 were collected, except for Central Java, in which data were available through September 2019. For the qualitative approach, 21 focus group discussions (FGDs) and 2 in-depth interviews were held among a total of 223 informants: 22 from Districts Health Department, 101 from Primary Health Care (PHC) facilities and 100 from POSBINDU cadres. The size of the FGDs was on average 10 (min 4, max 18). Verbatim transcripts of the FGD's were made for qualitative analyses.

Outcome

Missed opportunities in hypertension screening was quantified by the proportion of POSBINDU visitors getting the risk factors anamnesis, and measurement of anthropometric, blood pressure and cholesterol. We further explored the barriers of POSBINDU implementation using a qualitative approach.

Analyses

Statistical analyses were conducted using STATA, to calculate the proportion of activities and outcomes. We further conduct Chi-square, T-test, and ANOVA to assess the statistical significance of the differences. Content analysis was applied for the qualitative data to ascertain barriers for the POSBINDU implementation in Indonesia by two independent researchers. Parallel analyses were conducted to synthesize the findings from the quantitative and qualitative approaches.

Patient and Public Involvement

Patients or the public were not directly involved in the design, or conduct, or reporting, or dissemination plans of our research.

Results

Participation of community for hypertension screening in POSBINDU

Data from 114,581 POSBINDU visits (54,224 participants) were analyzed. The findings showed similar patterns in the districts and provinces: more female and elderly participants. Approximately 80% were

For peer review only - http://bmjopen.bmj.com/site/about/guidelines.xhtml

female participants, with the highest proportion of female participants in rural North Sumatra (95.5%). Meanwhile, in Java, a higher proportion of female participants were observed in urban areas (Table 1).

Table 1. Characteristics of POSBINDU Participants within the Three Provinces in Indonesia (POSBINDU Register, 2018-2019)

Characteristics	North Sumatra		East Java		Central Java		
	Rural	Urban	Rural	Urban	Rural	Urban	Total
Number of individuals	5,103	23,053	10,999	4,983	3,398	6,688	54,224
Number of PHC	11	23	29	9	11	17	100
Number of POSBINDU	38	283	38	27	27	62	475
Categorical (%, SE)							
Female	95.5 (0.3)	71.3 (0.4)	76.2 (0.3)	86.7 (0.5)	73.7 (0.8)	88.2 (0.4)	79.4 (0.2)
Age							
15-24	8.7 (0.4)	6.1 (0.3)	6.2 (0.2)	7.8 (0.4)	13.3 (0.7)	3.8 (0.3)	6.7 (0.1)
25-40	26.7 (0.7)	14.9 (0.4)	20.6 (0.3)	32.2 (0.7)	34.3 (1.0)	17.0 (0.6)	22.2 (0.2)
40-49	20.3 (0.6)	20.1 (0.4)	21.7 (0.3)	22.9 (0.6)	22.1 (0.8)	22.3 (0.6)	21.5 (0.2)
50-59	21.8 (0.6)	27.6 (0.5)	23.9 (0.3)	21.6 (0.6)	21.4 (0.8)	32.0 (0.7)	24.8 (0.2)
>60	22.4 (0.6)	31.3 (0.6)	27.6 (0.3)	12.5 (0.5)	8.9 (0.6)	24.9 (0.6)	24.7 (0.2)
Number of visits							
1 time	87.0 (0.4)	77.4 (0.3)	68.4 (0.3)	65.6 (0.7)	84.5 (0.6)	56.4 (0.6)	71.24 (0.1)
2-6 times	12.9 (0.5)	21.7 (0.3)	21.1 (0.2)	23.9 (0.6)	13.3 (0.6)	35.1 (0.6)	22.0 (0.1)
7-12 times	0.1 (0.0)	0.6 (0.1)	6.3 (0.2)	6.0 (0.3)	2.1 (0.2)	5.9 (0.3)	5.3 (0.1)
>12 times	0 (0.0)	0.2 (0.1)	4.1 (0.1)	4.5 (0.3)	0.1 (0.0)	2.6 (0.2)	1.4 (0.1)
Continuous (mean, SE)							
Age	46.4 (0.2)	51.7 (0.2)	49.7 (0.1)	43.4 (0.2)	41.0 (0.3)	50.4 (0.2)	48.6 (0.8)
Number of visits	1.2 (0.8)	1.4 (0.1)	2.5 (0.2)	2.7 (0.5)	1.4 (0.2)	2.5 (0.3)	2.1 (0.1)

Despite the relatively high missing information on age (n missing= 12,084, or 22.3%), we found that the participants were on average of older age, with roughly 50% of participants aged over 50 years old (Table 1). The highest proportion of participants >60 years old were observed in rural East Java (31.3%), with mean age of 51.7 years old. We measured the youngest POSBINDU participants in rural Central Java (mean age 41.0 years old).

In the span of the two years of secondary data collection, we found that, on average, the participants visit POSBINDU twice, with the lowest average of visits in North Sumatra (rounded to 1 visit/participant). Approximately 38,628 (71.2%) of participants visit POSBINDU once for two years, and 761 (1.4%) visits POSBINDU more than 12 times.

Table 2. Missed Opportunity in Hypertension Screening and Risk Factors Characteristics within POSBINDU Participants (POSBINDU Register, 2018-2019)

Characteristics	North Sumatra		East Java		Central Java		
	Rural %(SE)	Urban %(SE)	Rural %(SE)	Urban %(SE)	Rural %(SE)	Urban %(SE)	Total %(SE)

For peer review only - http://bmjopen.bmj.com/site/about/guidelines.xhtml

Missing Information in All Visits							
N	6,061	15,774	57,504	13,422	4,925	16,895	114,581
Personal history (complete)	99.3 (0.1)	92.6 (0.2)	67.9 (0.2)	42.5 (0.4)	62.1 (0.7)	95.2 (0.2)	73.8 (0.1)
Family history (complete)	99.4 (0.1)	92.6 (0.2)	65.1 (0.2)	39.7 (0.4)	61.6 (0.7)	95.9 (0.2)	72.1 (0.1)
Personal history (any)	88.2 (0.4)	88.5 (0.3)	56.4 (0.2)	17.2 (0.3)	54.2 (0.7)	91.0 (0.2)	62.9 (0.1)
Family history (any)	97.5 (0.2)	88.9 (0.3)	57.3 (0.2)	28.9 (0.4)	53.5 (0.7)	93.8 (0.2)	65.7 (0.1)
Height	42.7 (0.6)	19.0 (0.2)	15.6 (0.2)	20.7 (0.3)	30.6 (0.7)	23.3 (0.3)	19.9 (0.1)
Weight measurement	35.8 (0.6)	16.0 (0.2)	18.8 (0.2)	5.6 (0.2)	8.9 (0.4)	12.3 (0.3)	16.4 (0.1)
Waist circumference	49.5 (0.6)	36.0 (0.3)	15.6 (0.2)	12.7 (0.2)	36.9 (0.6)	63.6 (0.4)	27.8 ().1)
Blood pressure	1.8 (0.2)	5.8 (0.2)	9.8 (0.1)	4.6 (0.2)	4.2 (0.3)	6.3 (0.2)	7.4 (0.1)
Blood cholesterol	87.0 (0.4)	80.1 (0.3)	81.9 (0.2)	97.4 (0.1)	91.1 (0.4)	82.0 (0.3)	84.2 (0.1)
**Missing Information in First Visits**							
N	5,103	23,053	10,999	4,983	3,398	6,688	54,224
Personal history (complete)	99.2 (0.1)	89.9 (0.3)	72.8 (0.3)	35.1 (0.7)	49.3 (0.9)	92.6 (0.3)	76.3 (0.2)
Family history (complete)	99.3 (0.1)	89.8 (0.3)	71.3 (0.3)	41.4 (0.7)	50.9 (0.9)	93.5 (0.3)	76.4 (0.2)
Personal history (any)	88.2 (0.5)	85.1 (0.3)	58.3 (0.3)	15.3 (0.5)	40.1 (0.8)	86.9 (0.4)	68.3 (0.2)
Family history (any)	97.3 ().2)	85.2 (0.3)	60.6 (0.3)	30.0 (0.6)	40.1 (0.8)	90.5 (0.4)	65.0 (0.2)
Height	41.8 (0.7)	19.0 (0.4)	14.8 (0.2)	21.1 (0.6)	20.4 (0.7)	23.0 (0.5)	20.1 (0.2)
Weight	35.3 (0.3)	15.0 (0.3)	16.7 (0.2)	47.6 (0.3)	7.2 ().4)	12.9 (0.4)	15.9 (0.1)
Waist circumference	48.9 ().7)	33.6 (0.4)	11.1 (0.2)	12.9 (0.5)	23.0 ().7)	66.1 (0.6)	26.9 (0.2)
Blood pressure	1.7 (0.2)	5.4 (0.2)	4.9 (0.1)	5.0 (0.3)	3.2 (0.3)	7.0 (0.3)	4.9 (0.1)
Blood cholesterol	86.1 (0.5)	75.7 (0.4)	76.6 (0.3)	97.9 (0.2)	92.3 (0.4)	79.1 (0.5)	80.6 (0.2)
**Risk Factors Screening in All Visits**							
BMI							
Normal	48.3 (0.9)	48.2 (0.5)	51.3 (0.2)	44.6 (0.5)	52.3 (0.9)	46.4 (0.4)	49.3 (0.2)
Underweight	4.9 (0.4)	4.5 (0.2)	8.0 (0.1)	4.5 (0.2)	9.1 (0.5)	3.8 (0.2)	6.3 (0.1)
Overweight	31.9 (0.8)	34.4 (0.4)	30.8 (0.2)	34.7 (0.4)	29.1 (0.8)	34.2 (0.4)	32.3 (0.2)
Obese	14.9 (0.6)	12.9 (0.3)	10.0 (0.3)	16.3 (0.4)	9.4 (0.5)	15.7 (0.3)	12.1 (0.1)
Hypertension	35.4 (0.6)	28.0 (0.4)	42.5 (0.2)	33.7 (0.4)	25.6 (0.6)	35.9 (0.4)	37.2 (0.1)
**Risk Factors Screening in First Visits**							
BMI							
Normal	49.3 (0.9)	48.0 (0.5)	51.5 (0.4)	45.3 (0.8)	52.4 (1.0)	44.4 (0.7)	49.3 (0.2)
Underweight	4.8 (0.4)	4.5 (0.2)	7.7 (0.2)	5.1 (0.4)	10.1 (0.6)	4.2 (0.3)	6.3 (0.1)
Overweight	32.0 (0.9)	34.2 (0.5)	30.4 (0.3)	33.3 (0.8)	28.7 (0.9)	35.1 (0.7)	32.0 (0.2)
Obese	13.9 (0.6)	13.3 (0.4)	10.4 (0.2)	16.3 (0.6)	8.8 (0.5)	16.3 (0.5)	12.4 (0.2)
Hypertension	34.5 (0.7)	28.5 (0.4)	40.5 (0.3)	31.6 (0.7)	25.1 (0.8)	37.9 (0.6)	35.3 (0.2)

Within province, rural-urban comparisons are significant at 0.05
Between provinces comparisons are significant at 0.05

We further observed the relatively high missing information for hypertension screening across the districts, with the following general pattern; First, a relatively high proportion of missing information concerning the personal and family history, with East Java having the lowest proportion. Second, a

For peer review only - http://bmjopen.bmj.com/site/about/guidelines.xhtml

relatively lower missing data on anthropometric measurements (less than 50%). Third, we found the highest proportion of available data for blood pressure measurements in all the seven districts. Last, our analysis identified higher missing values for blood cholesterol measurements (84.2%). For all measurements, there were significant differences between the three provinces, as well as between the rural and urban areas within the provinces (Table 2).

Based on available data, we found that obesity seems to be more prevalent in urban areas in Java, but relatively similar in North Sumatra. In contrast, hypertension was more prevalent in a rural area for East Java and North Sumatra but was more common in urban districts of Central Java (Table 2). However, these data should be interpreted cautiously due to the relatively high missing data on the measurements.

Barriers for the screening of hypertension in POSBINDU

The qualitative data supported the quantitative finding about lacking participation of male and younger population to POSBINDU. In the FGDs, cadres and health officials stated the barriers for male and younger participants to attend POSBINDU, including the inconvenience of POSBINDU schedule, as well as low awareness for hypertension screening (Table 3).

Table 3. Qualitative analyses of Focus Group Discussion amongst POSBINDU Cadres, Primary Health Care and Health Department Officials

Themes	Category	Codes
Suboptimal target population and gap in policy	Participants' characteristics	Younger adults rarely participate
		Lack of male participants
	Barrier to participations	Schedule incompatibility
		Low awareness for screening
		Lack of role model for screening
	Ineffective policy and coordination	Lack of prioritization for NCD
		Implementation gap of national policy/program at the local level
		The need for coordination with different stakeholders
		The need for coordination among NCD-related programs
Lack of human resources in terms of capability and quantity for hypertension screening	Cadres have multiple tasks, with time constraints	POSBINDU cadres often have to multitask and handling other community programs
		Cadres are volunteers with other obligations
	Cadres' competencies	Lack of knowledge on hypertension and other NCD
		Lack of ability to conduct measurements and provide health education
		Lack of ability to conduct recording and reporting
	Lack of NCD program officers for supervision and reporting	Lack of NCD program officers at PHC
		Most program officers are responsible for multiple tasks/programs
		Lack of reporting officers
	Provision of Referral Counselling	The participant with hypertension is not always referred to PHC

For peer review only - http://bmjopen.bmj.com/site/about/guidelines.xhtml

		Lack of counseling to participants before the referral made
		POSBINDU has referral form, but rarely used
		Treatment for the referral is covered by their health insurance
Lack of resources for hypertension screening and prevention	Equipment for hypertension screening	The equipment is sometimes incomplete
		Equipment maintenance is inadequate
		Limited logistics for cholesterol measurement
	Lack of budget	POSBINDU is funded by the government, stakeholder (private sectors) or community
		Lack of budget for POSBINDU activities
		Lack of budget for cadres training and incentives
	Health education material	Lack of health education materials
	Infrastructure for recording and reporting	Not all cadres have laptops
		Limited internet connection in some areas
		Most POSBINDU stations use manual reporting
Time constraints for implementation based on MOH standard	The complexity of activities and time limitation	The time required for examination is too long
		Too many information needs to be asked and filled out
		The referral form is rarely used
	The complexity of reporting forms	Many forms need to be filled, while time is limited
		A simplified form in checklist format is preferred

The need for role model from community leader to improve participation and the barrier for participation, particularly among males is highlighted by these quotes:

" Yes, we don't have a lot of men (participants), because they are working " (Cadre, FGD#21)

" In our POSBINDU*, the awareness for early screening is still low. Only several people come (to* POSBINDU*), younger people don't want to come because (*POSBINDU *is conducted) during working days " (Cadre, FGD#3)*

" …Socialization for this (hypertension screening) is needed, often, the community leader in our area don't want to participate because they are afraid to be screened" (Cadre, FGD#2)

Lack of priority and overlap of NCD-related programs also contribute to a suboptimal target population of POSBINDU, as illustrated by the following quote:

" (NCD) is not a priority program, hence, there's a lack of commitment between the superior (health department) with the program officials, for example." (Health official, FGD#7) "…(different department in) the Ministry of Health focus on specific diseases, such as diabetes and cancer… However, in the community, (the programs) become general. (We) run Polindes, (Posyandu) Lansia, POSBINDU, School Health Program. In my opinion, the regulation is rigid and detailed, but the implementation is mixed (overlap). If we want to give optimum results, it takes efforts." (Health official, FGD#10).

Several barriers for implementation were revealed. The cadres and health officers often have to run several different programs. The FGDs also revealed a lack of capability of cadres to conduct

For peer review only - http://bmjopen.bmj.com/site/about/guidelines.xhtml

measurements for hypertension screening, providing health education, and also conducting the recording and reporting of the POSBINDU activities and measurements. The informants also mentioned a lack of resources, including budget, equipment, and logistics to conduct all the measurements.

> *" One person can hold 5 positions in PHC activities… POSBINDU cadres, Posyandu Lansia cadres, and other programs.. " (Cadre, FGD#11)*

> *" (cadres of) Posbindu do not have laptop nor cell phone for the reporting application (of* POSBINDU*), hence, we report to PHC manually" (Cadre, FGD#14)*

The barriers also include the complexities of the activities and measurements, as well as extensive reporting forms, which require a long time to be completed.

> *" …it takes a long time, because of the measurements and stages (of* POSBINDU *activities) " (PHC officer, FGD#8)*

> *" …*POSBINDU *report is too time-consuming, because it is long (detail), including identity, cell phone number, address, and others… and it has to be filled out every month." (PHC officer, FGD#6)*

Interestingly, in several districts, we found the implementation of mobile POSBINDU, moving from one community to the other within the same subdistricts.

> *"Our* POSBINDU *is mobile, we have ten communities, so every week, we move from one community to the next, focusing on people 15-59 years old." (Cadre, FGD#18)*

We further synthesized the quantitative and qualitative results. We categorized the barriers into three main parts: 1) input, reflecting the target population/coverage of POSBINDU, 2) process, describing the implementation of POSBINDU activities, and 3) output, reflecting the recording and reporting process of POSBINDU (Figure 1). Results show that in both approaches we found lacking participation of male and younger people in POSBINDU. Lack of priority for NCD screening and ineffective coordination among stakeholders, combined with lack of awareness and access might attribute to this finding. The high missed opportunity, particularly in history taking and measurements, were likely due to the complexity of the activities/measurements, as well as lack of resources. The high missing data also stem from the complexity of the forms and lack of capability for online reporting.

Insert Figure 1 here

Discussion

In this study, we revealed missed opportunities in input, activities, and output of POSBINDU implementation. Several contextual barriers were identified. The suboptimal coverage was possibly due to lack of priority for NCD screening, lack of awareness and access, and overlap of NCD-related program. The suboptimal activities and reporting were likely caused by a lack of resources, as well as limited time to perform the complexities of activities and reporting according to MOH guideline.

The missed opportunity to screen male and younger population that we found in this study is particularly concerning. Although the prevalence is lower than of the older population, hypertension prevalence among young Indonesian is still relatively high (28%).[22] While the target population of POSBINDU is listed as those 15 years or older, the elderly are usually targeted in Posyandu Lansia, a community-based screening and management for the elderly population.[23] Awareness is also lower in male and younger adults, signaling the need to screen this population.[3] Ideally, POSBINDU

For peer review only - http://bmjopen.bmj.com/site/about/guidelines.xhtml

becomes the "gatekeeper" for screening in the community. Hypertensive and diabetic patients were then referred to PHC and joined Prolanis, a community-based activity funded by the health insurance program, for management of chronic diseases patients.[12]

Furthermore, with a lack of male participation, POSBINDU is missing one of the key target populations for risk factors screening: smokers. Analysis of a national survey in 2014 reported 32% prevalence of smoking, with approximately 40% of males aged 15-55 years old and 14% of male adolescents are current smokers.[24–26] Further, 20% of Indonesia's total chronic diseases are attributed to smoking, with hypertension as the highest proportion.[27] Screening for hypertension and its risk factors earlier, combined with lifestyle-based interventions effectively avoid future complications.[28,29]

We also revealed the need to prioritize and reorganize the current NCD-related programs, to address the suboptimal coverage and the overlap. An example of the gap between the national recommendation and local implementation is reflected in the coordination of existing NCD-related programs: POSBINDU, Posyandu Lansia, and PANDU PTM. In the MOH, the PANDU PTM and POSBINDU are regulated under the Directorate for Disease Management, while Posyandu Lansia is under the Directorate of Public Health. Despite the different directorates, the implementation at community level is often conducted simultaneously and often overlap. Reporting, however, is conducted separately. Hence, as previous studies have noted, we also recommend the need of comprehensive and coordinated NCD prevention program in Indonesia.[30–32]

The relatively high missed opportunity in hypertension screening portrays suboptimal implementation of POSBINDU. This can be caused by a lack of recording and reporting (monitoring and evaluation fidelity) or lack of measurement (implementation fidelity). Lack of human resources might contribute to the suboptimal implementation of POSBINDU. Our findings revealed the need to train cadres to improve their skills and efficiency in conducting the measurements and history taking. This is in line with findings from Meinema et al (2017 and Abdell-All et al (2018)[33,34]. Our findings also imply the complexities of the activities and reporting of POSBINDU which lead to ineffective implementation. It is important to ensure that valuable screening information can be recorded and followed up, for better intervention. A simplified screening program with integrated reporting is needed.

In this study, we also discovered lack of financial and equipment as barrier to POSBINDU implementation. The integration of POSBINDU and PANDU PTM to the national health insurance scheme might be important to ensure the sustainability of funding for the program. Integration of POSBINDU into the national health insurance can also improve participation of the working population, most of whom are covered by the national health insurance.[35] Previous studies have reported an increase in uptake of service by health insurance membership.[36–38]

Based on our findings, we identified two main areas that needs to be improved: coverage and implementation of POSBINDU. To improve coverage of POSBINDU, there are two important steps that we recommend. First, an integrated approach with collaboration amongst different programs and directorates to reduce the overlap and simplify the POSBINDU implementation at the PHC and community level. PANDU PTM as the adaptation of WHO PEN,[39] needs to be implemented in a wider scale. Second, redirecting the target population of hypertension screening, to cover also younger and male population. A workplace-based screening program which can address the barriers identified in the qualitative findings is recommended.[40,41] For this younger population, the use of mobile technology for monitoring of risk factors and measurement might be effective. Previous studies have reported the effectiveness of mobile health for hypertension screening and risk stratification.[42,43]

For peer review only - http://bmjopen.bmj.com/site/about/guidelines.xhtml

To improve the implementation and components of POSBINDU activities, a simplified algorithm to screen and refer the target population is needed. The algorithm needs to be developed both in the electronic format and manual format to address the different capabilities of community cadres and resources in the community. Simplifying the program and reporting systems will also reduce the workload of PHC and district health officials. Further, a clear algorithm for the management of "screened" cases to PHC is important. The readiness of the PHCs also needs to be improved to adequately manage the potential surge in referred cases. Lastly, there is a need to integrate hypertension and NCD screening program into the national health insurance system. Hence, ensuring the sustainability of funding and resources of the program. With these approaches, comprehensive screening for hypertension and NCD along the continuum of care might be more effective.

This study has several limitations. First, the proportion of our measures are not reflective for the whole target population of POSBINDU, since the participants were mostly female and of older age. The characteristics of our sample, which are generally older with a higher proportion of females, drive the proportion higher than that of the general population in Indonesia. However, this study reflects the current participants of POSBINDU. Second, we used a secondary data collection by POSBINDU cadres, the missingness that we presented in this study probably stem from two main sources: lacking in reporting or a true lack in measurement/activities. Nevertheless, both the activities and reporting are important in NCD screening, particularly in the follow-up.

Despite the limitation, there are several strengths of the study: First, to our knowledge, this was the first relatively large evaluation of POSBINDU. Second, the use of a mixed-methods study design. Therefore, providing more comprehensive information on POSBINDU implementation. Third, the study also investigates the contextual factors that should be addressed in the improvement of the community-based hypertension screening program in Indonesia.

Conclusion

This study showed the missed opportunities of POSBINDU for hypertension screening in Indonesia. The barriers include a lack priority for NCDs, lack of awareness and access for subpopulation, and several implementation barriers: capability, resources, and protocols. An innovative approach to simplify and improve the capacity of POSBINDU is in preparation to optimize the screening and linkage to care of hypertension in Indonesia. This study provides evidence-based recommendations in improving the current implementation of POSBINDU, in the Indonesian context.

Data Statement

The de-identified data from this study is available upon request to the corresponding or first author pending thorough review of request and adherence to the Indonesian government regulation on data sharing.

Author Contributions

VW, AP, RFP, EP, YM, JK and JLD contributed in the design of the study. VW, A, RFP, EP, YM, B, and M participate actively in study implementation, including in data collection. VW, YM, S, and RFP analyzed the quantitative data. AP, EP, and B analyzed the qualitative data. VW, AP, RFP, S, and YM draft the manuscript with all co-authors revised critically. All authors read and approved the final manuscript.

Funding

This work was supported by the Horizon 2020 research program of the European Union, with grant number 825026.

For peer review only - http://bmjopen.bmj.com/site/about/guidelines.xhtml

Conflict of Interest

None declared

License

The Corresponding Author has the right to grant on behalf of all authors and does grant on behalf of all authors, an exclusive licence (or non exclusive for government employees) on a worldwide basis to the BMJ Publishing Group Ltd ("BMJ"), and its Licencees to permit this article (if accepted) to be published in The BMJ's editions and any other BMJ products and to exploit all subsidiary rights, as set out in our licence.

Acknowledgment

This study was co-financed by the Horizon 2020 research program of the European Union, under grant 825026 Scaling Up NCD interventions in South East Asia.

References

1 Basu S, Millett C. Social epidemiology of hypertension in middle-income countries: Determinants of prevalence, diagnosis, treatment, and control in the WHO SAGE study. *Hypertension* 2013;**62**:18–26. doi:10.1161/HYPERTENSIONAHA.113.01374

2 Mills KT, Bundy JD, Kelly TN, *et al.* Global disparities of hypertension prevalence and control. *Circulation* 2016;**134**:441–50. doi:10.1161/CIRCULATIONAHA.115.018912

3 Hussain MA, Al Mamun A, Reid C, *et al.* Prevalence, awareness, treatment and control of hypertension in Indonesian adults aged ≥40 years: Findings from the Indonesia Family Life Survey (IFLS). *PLoS One* 2016;**11**:1–16. doi:10.1371/journal.pone.0160922

4 Peltzer K, Pengpid S. The Prevalence and Social Determinants of Hypertension among Adults in Indonesia: A Cross-Sectional Population-Based National Survey. *Int J Hypertens* 2018;**2018**. doi:10.1155/2018/5610725

5 Purnamasari D. The Emergence of Non-communicable Disease in Indonesia. *Acta Med Indones* 2018;**50**:273–4.http://www.ncbi.nlm.nih.gov/pubmed/30630990

6 Vos T, Abajobir AA, Abbafati C, *et al.* Global, regional, and national incidence, prevalence, and years lived with disability for 328 diseases and injuries for 195 countries, 1990-2016: A systematic analysis for the Global Burden of Disease Study 2016. *Lancet* 2017;**390**:1211–59. doi:10.1016/S0140-6736(17)32154-2

7 Gaziano T. Prevention and treatment of chronic diseases in developing countries. 2011;:1–20.http://www.un.org/esa/population/publications/expertpapers/2011-2-gaziano.pdf

8 Allen L, Williams J, Townsend N, *et al.* Socioeconomic status and non-communicable disease behavioural risk factors in low-income and lower-middle-income countries: a systematic review. *Lancet Glob Heal* 2017;**5**:e277–89. doi:10.1016/S2214-109X(17)30058-X

9 Rosengren A, Smyth A, Rangarajan S, *et al.* Socioeconomic status and risk of cardiovascular disease in 20 low-income, middle-income, and high-income countries: the Prospective Urban Rural Epidemiologic (PURE) study. *Lancet Glob Heal* 2019;**7**:e748–60. doi:10.1016/S2214-109X(19)30045-2

10 WHO. Package of Essential Noncommunicable (PEN) Disease Interventions for Primary Health Care. *Geneva World Heal Organ* 2010;:1–66.papers2://publication/uuid/FC98856B-4122-4325-826F-20FA6B89DEBD

11 Ferdianto A, Tamtomo DG, Sulaeman ES. Does the Integrated Health Post have Contextual

For peer review only - http://bmjopen.bmj.com/site/about/guidelines.xhtml

Effect on Tertiary Preventive Behavior among Hypertensive Patients? A Multilevel Analysis Evidence from Surakarta. *J Heal Promot Behav* 2019;**4**:224–34. doi:10.26911/thejhpb.2019.04.03.07

12 Khoe LC, Wangge G, Soewondo P, *et al.* The implementation of community-based diabetes and hypertension management care program in Indonesia. *PLoS One* 2020;**15**:1–9. doi:10.1371/journal.pone.0227806

13 Yamada M, Hapsari ED, Matsuo H. Behaviors toward noncommunicable diseases prevention and their relationship with physical health status among community-dwelling, middleaged and older women in Indonesia. *Int J Environ Res Public Health* 2020;**17**. doi:10.3390/ijerph17072332

14 Maharani A, Sujarwoto, Praveen D, *et al.* Cardiovascular disease risk factor prevalence and estimated 10-year cardiovascular risk scores in Indonesia: The SMARThealth Extend study. *PLoS One* 2019;**14**:1–13. doi:10.1371/journal.pone.0215219

15 Turana Y, Tengkawan J, Soenarta AA. Asian management of hypertension: Current status, home blood pressure, and specific concerns in Indonesia. *J Clin Hypertens* 2020;**22**:483–5. doi:10.1111/jch.13681

16 Scheltens T, Bots ML, Numans ME, *et al.* Awareness, treatment and control of hypertension: The 'rule of halves' in an era of risk-based treatment of hypertension. *J Hum Hypertens* 2007;**21**:99–106. doi:10.1038/sj.jhh.1002123

17 Chen R, Tunstall-Pedoe H, Morrison C, *et al.* Trends and social factors in blood pressure control in Scottish MONICA surveys 1986-1995: The rule of halves revisited. *J Hum Hypertens* 2003;**17**:751–9. doi:10.1038/sj.jhh.1001612

18 Alfiyah A, Pujiyanto P. an Analysis on the Implementation of the Integrated Guidance Post (Posbindu) Activities for Non-Communicable Diseases At Bogor City in 2018. *J Indones Heal Policy Adm* 2019;**4**:11–5. doi:10.7454/ihpa.v4i1.2388

19 Ministry of Health (MOH) - Kementerian Kesehatan. Petunjuk Teknis Pos Pembinaan Terpadu Penyakit Tidak Menular (Posbindu PTM). 2012. http://p2ptm.kemkes.go.id/uploads/2016/10/Petunjuk-Teknis-Pos-Pembinaan-Terpadu-Penyakit-Tidak-Menular-POSBINDU-PTM-2013.pdf

20 Moeloek NF. Indonesia national health policy in the transition of disease burden and health insurance coverage. *Med J Indones* 2017;**26**:3–6. doi:10.13181/mji.v26i1.1975

21 Ministry of Health (MOH) - Kementerian Kesehatan. Riskesdas Main Results 2018. 2018. https://www.litbang.kemkes.go.id/hasil-utama-riskesdas-2018/

22 Sudharsanan N, Geldsetzer P. Impact of coming demographic changes on the number of adults in need of care for hypertension in Brazil, China, India, Indonesia, Mexico, and South Africa: A modeling study. *Hypertension* 2019;**73**:770–6. doi:10.1161/HYPERTENSIONAHA.118.12337

23 Rahmawati R, Bajorek B V. Access to medicines for hypertension: A survey in rural Yogyakarta province, Indonesia. *Rural Remote Health* 2018;**18**. doi:10.22605/RRH4393

24 Amalia B, Cadogan SL, Prabandari YS, *et al.* Socio-demographic inequalities in cigarette smoking in Indonesia, 2007 to 2014. *Prev Med (Baltim)* 2019;**123**:27–33. doi:10.1016/j.ypmed.2019.02.025

25 Sujarwoto S. Sleep Disturbance in Indonesia: How Much Does Smoking Contribute? *Behav*

For peer review only - http://bmjopen.bmj.com/site/about/guidelines.xhtml

Sleep Med 2019;**00**:1–14. doi:10.1080/15402002.2019.1682584

26 Kusumawardani N, Tarigan I, Suparmi, *et al.* Socio-economic, demographic and geographic correlates of cigarette smoking among Indonesian adolescents: results from the 2013 Indonesian Basic Health Research (RISKESDAS) survey. *Glob Health Action* 2018;**11**. doi:10.1080/16549716.2018.1467605

27 Kristina SA, Endarti D, Wiedyaningsih C, *et al.* Health Care Cost of Noncommunicable Diseases Related to Smoking in Indonesia, 2015. *Asia-Pacific J Public Heal* 2018;**30**:29–35. doi:10.1177/1010539517751311

28 Saif-Ur-Rahman KM, Hasan M, Hossain S, *et al.* Non-pharmacological interventions for the prevention of hypertension in low-income and middle-income countries: Protocol for a systematic review and meta-analysis. *BMJ Open* 2018;**8**:6–11. doi:10.1136/bmjopen-2017-020724

29 Pantell MS, Prather AA, Downing JM, *et al.* Association of Social and Behavioral Risk Factors With Earlier Onset of Adult Hypertension and Diabetes. *JAMA Netw open* 2019;**2**:e193933. doi:10.1001/jamanetworkopen.2019.3933

30 Kusuma D, Kusumawardani N, Ahsan A, *et al.* On the verge of a chronic disease epidemic: Comprehensive policies and actions are needed in Indonesia. *Int Health* 2019;**11**:422–4. doi:10.1093/inthealth/ihz025

31 Mboi N, Murty Surbakti I, Trihandini I, *et al.* On the road to universal health care in Indonesia, 1990–2016: a systematic analysis for the Global Burden of Disease Study 2016. *Lancet* 2018;**392**:581–91. doi:10.1016/S0140-6736(18)30595-6

32 Jayanna K, Swaroop N, Kar A, *et al.* Designing a comprehensive Non-Communicable Diseases (NCD) programme for hypertension and diabetes at primary health care level: Evidence and experience from urban Karnataka, South India. *BMC Public Health* 2019;**19**:1–12. doi:10.1186/s12889-019-6735-z

33 Meinema JG, Haafkens JA, Jaarsma DADC, *et al.* Development and evaluation of a culturally appropriate hypertension education (CAHE) training program for health care providers. *PLoS One* 2017;**12**:1–13. doi:10.1371/journal.pone.0178468

34 Abdel-All M, Thrift AG, Riddell M, *et al.* Evaluation of a training program of hypertension for accredited social health activists (ASHA) in rural India. *BMC Health Serv Res* 2018;**18**:1–11. doi:10.1186/s12913-018-3140-8

35 Ng JYS, Ramadani RV, Hendrawan D, *et al.* National Health Insurance Databases in Indonesia, Vietnam and the Philippines. *PharmacoEconomics - Open* 2019;**3**:517–26. doi:10.1007/s41669-019-0127-2

36 Erlangga D, Ali S, Bloor K. The impact of public health insurance on healthcare utilisation in Indonesia: evidence from panel data. *Int J Public Health* 2019;**64**:603–13. doi:10.1007/s00038-019-01215-2

37 Sharma M, Teerawattananon Y, Luz A, *et al.* Institutionalizing Evidence-Informed Priority Setting for Universal Health Coverage: Lessons From Indonesia. *Inq (United States)* 2020;**57**. doi:10.1177/0046958020924920

38 Vidyattama Y, Miranti R, Resosudarmo BP. The Role of Health Insurance Membership in Health Service Utilisation in Indonesia. *Bull Indones Econ Stud* 2014;**50**:393–413. doi:10.1080/00074918.2014.980380

For peer review only - http://bmjopen.bmj.com/site/about/guidelines.xhtml

39 Ministry of Health (MOH) - Kementerian Kesehatan. Pandu PTM A Community on intervention model and control's of NCD's. 2017.

40 Wang Z, Wang X, Shen Y, *et al.* Effect of a Workplace-Based Multicomponent Intervention on Hypertension Control: A Randomized Clinical Trial. *JAMA Cardiol* 2020;**5**:567–75. doi:10.1001/jamacardio.2019.6161

41 Reif J, Chan D, Jones D, *et al.* Effects of a Workplace Wellness Program on Employee Health, Health Beliefs, and Medical Use: A Randomized Clinical Trial. *JAMA Intern Med* 2020;**180**:952–60. doi:10.1001/jamainternmed.2020.1321

42 Patel A, Praveen D, Maharani A, *et al.* Association of Multifaceted Mobile Technology-Enabled Primary Care Intervention with Cardiovascular Disease Risk Management in Rural Indonesia. *JAMA Cardiol* 2019;**4**:978–86. doi:10.1001/jamacardio.2019.2974

43 Persell SD, Peprah YA, Lipiszko D, *et al.* Effect of Home Blood Pressure Monitoring via a Smartphone Hypertension Coaching Application or Tracking Application on Adults With Uncontrolled Hypertension: A Randomized Clinical Trial. *JAMA Netw open* 2020;**3**:e200255. doi:10.1001/jamanetworkopen.2020.0255

For peer review only

For peer review only - http://bmjopen.bmj.com/site/about/guidelines.xhtml

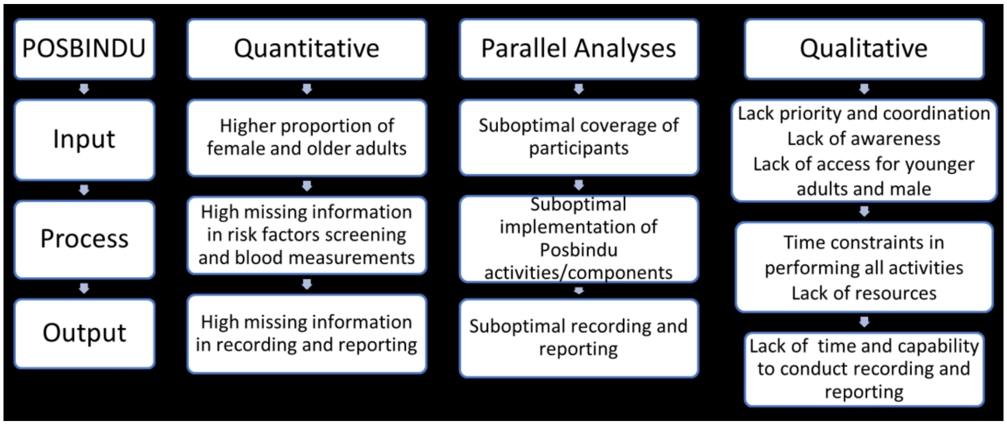

Figure 1. Synthesis of the Quantitative and Qualitative Findings

BMJ Open

Missed Opportunities in Hypertension Risk Factors Screening in Indonesia: A Mixed-methods Evaluation of Integrated Health Post (POSBINDU) Implementation

Journal:	*BMJ Open*
Manuscript ID	bmjopen-2021-051315.R1
Article Type:	Original research
Date Submitted by the Author:	17-Nov-2021
Complete List of Authors:	Widyaningsih, Vitri; Universitas Sebelas Maret, Public Health<br>Febrinasari, Ratih; Universitas Sebelas Maret, Pharmacology<br>Pamungkasari, Eti; Universitas Sebelas Maret, Public Health<br>Mashuri, Yusuf; Universitas Sebelas Maret, Parasitology<br>Sumardiyono, Sumardiyono; Universitas Sebelas Maret, Public Health<br>Balgis, Balgis; Universitas Sebelas Maret, Public Health<br>Koot, Jaap; University Medical Centre Groningen, Department of Health Sciences<br>Landsman-Dijkstra, Jeanet; University Medical Centre Groningen, Department of Health Sciences<br>Probandari, Ari; Universitas Sebelas Maret,
**Primary Subject Heading**:	Public health
Secondary Subject Heading:	Global health
Keywords:	Hypertension < CARDIOLOGY, International health services < HEALTH SERVICES ADMINISTRATION & MANAGEMENT, PUBLIC HEALTH, Quality in health care < HEALTH SERVICES ADMINISTRATION & MANAGEMENT

SCHOLARONE™
Manuscripts

I, the Submitting Author has the right to grant and does grant on behalf of all authors of the Work (as defined in the below author licence), an exclusive licence and/or a non-exclusive licence for contributions from authors who are: i) UK Crown employees; ii) where BMJ has agreed a CC-BY licence shall apply, and/or iii) in accordance with the terms applicable for US Federal Government officers or employees acting as part of their official duties; on a worldwide, perpetual, irrevocable, royalty-free basis to BMJ Publishing Group Ltd ("BMJ") its licensees and where the relevant Journal is co-owned by BMJ to the co-owners of the Journal, to publish the Work in this journal and any other BMJ products and to exploit all rights, as set out in our licence.

The Submitting Author accepts and understands that any supply made under these terms is made by BMJ to the Submitting Author unless you are acting as an employee on behalf of your employer or a postgraduate student of an affiliated institution which is paying any applicable article publishing charge ("APC") for Open Access articles. Where the Submitting Author wishes to make the Work available on an Open Access basis (and intends to pay the relevant APC), the terms of reuse of such Open Access shall be governed by a Creative Commons licence – details of these licences and which Creative Commons licence will apply to this Work are set out in our licence referred to above.

Other than as permitted in any relevant BMJ Author's Self Archiving Policies, I confirm this Work has not been accepted for publication elsewhere, is not being considered for publication elsewhere and does not duplicate material already published. I confirm all authors consent to publication of this Work and authorise the granting of this licence.

For peer review only

Missed Opportunities in Hypertension Risk Factors Screening in Indonesia: A Mixed-methods Evaluation of Integrated Health Post (POSBINDU) Implementation

Vitri Widyaningsih[1] , Ratih Puspita Febrinasari[1], Eti Poncorini Pamungkasari [1], Yusuf Ari Mashuri[1], Sumardiyono[1], Balgis[1], Jaap Koot[2], Jeanet Landsman-Dijkstra[2], Ari Probandari [1] on behalf of Scaling Up Non-Communicable Disease Intervention in South East Asia (SUNISEA) Project

[1]Faculty of Medicine, Universitas Sebelas Maret, Surakarta, Indonesia

[2] University of Groningen, University Medical Center Groningen, Department of Health Sciences, The Netherlands

Corresponding author: Prof. dr. Ari Natalia Probandari, MPH., Ph.D. Faculty of Medicine Universitas Sebelas Maret Surakarta, 57126, Central Java, Indonesia ari.probandari@staff.uns.ac.id

Phone: (0271) 646994

Abstract

Objectives: To assess the implementation and contextual barriers of POSBINDU, a community-based activity focusing on screening of Non-Communicable Diseases (NCDs), mainly hypertension and diabetes, in Indonesia.

Design: This was a concurrent mixed-methods study, with a cross-sectional analysis of secondary data and Focus Group Discussions on stakeholder of POSBINDU.

Setting: The study was conducted in seven districts in three provinces in Indonesia, with approximately 50% of the primary health care (PHC) were selected as areas for data collection (n PHC=100).

Participants: From 475 POSBINDU sites, we collected secondary data from 54,224 participants. For the qualitative approach, 21 focus group discussions (FGDs) and 2 in-depth interviews were held among a total of 223 informants.

Primary outcomes and measures: Proportion of POSBINDU visitors getting the hypertension screening and risk factors' assessment, and barriers of POSBINDU implementation.

Results: Out of the 114,581 POSBINDU visits by 54,224 participants, most (80%) were female and adults over 50 years old (50%) showing a suboptimal coverage of male and younger adults. Approximately 95.1% of visitors got their blood pressure measured during their first visit; 353% of whom had elevated blood pressure. Less than 25% of the visitors reported to be interviewed for NCDs risk factors during their first visit, less than 80% had anthropometric measurements, and less than 15% had blood cholesterol examinations. We revealed lack of resources and limited time to perform the complexities of activities and reporting as main barrier for effective hypertension screening in Indonesia.

Conclusions: This study showed missed opportunities in hypertension risk factors screening in Indonesia. The barriers include a lack of access and implementation barriers (capability, resources, and protocols).

Keywords: Non-communicable diseases, hypertension, screening, community-based program

Article Summary

Findings

For peer review only

For peer review only - http://bmjopen.bmj.com/site/about/guidelines.xhtml

In a mixed-methods study, we found suboptimal implementation of POSBINDU which reflected the missed opportunities in screening for hypertension and its risk factors in Indonesia. Several barriers include suboptimal coverage, complexities of activities and overlap between different NCD-related programs, and lack of resources.

Implications

There is a need to improve coverage and implementation of POSBINDU for screening for hypertension and its risk factors. An integrated approach to improve the implementation of hypertension screening, from guidelines to practice is crucial.

Strengths and Limitations of this study

- This was a relatively large evaluation of POSBINDU in Indonesia, with almost two years of data.
- The findings from mixed-methods study provide more comprehensive information on POSBINDU implementation
- Information on the contextual factors of POSBINDU implementation can provide insights into steps to improve POSBINDU in the communities .
- The use of secondary data poses variations in blood pressure and anthropometrics measurements.
- The study limitation also includes the difficulty in differentiating whether the missed reporting was due to lack of activities or lack of reporting. Nevertheless, both the activities and reporting are important in NCDs screening, particularly in the follow up.

Introduction

The increasing trends of Non-Communicable Diseases (NCDs) in the world, including Indonesia, require targeted and specific primary and secondary prevention.[1,2] Hypertension, one of the most common NCDs, has a relatively high (33.4%) prevalence in Indonesia.[3][4] This figure is estimated to increase even further with the changing (more sedentary) lifestyle, unhealthy diet, rising prevalence of obesity, and the increasing life expectancy.[5] In 2015, hypertension attributed to 41% of all disability-adjusted life-years (DALYs) lost, and was the leading risk factor for cardiovascular diseases.[6] Economically, hypertension accounts for $370 billion in medical costs per year worldwide.[7] Major modifiable risk factors for NCDs include smoking, alcohol consumption, unhealthy diet and obesity, and a sedentary lifestyle.[8,9] With the heavy burden and the economic cost of this disease, primary and secondary prevention for hypertension and its risk factors become very important.

In 2010, the WHO has recommended the implementation of Package of Essential Interventions for Non-Communicable (PEN) Diseases for low- and middle-income countries.[10] In response, the Ministry of Health (MOH) in Indonesia launched the Integrated Health Post (POSBINDU), as part of the PEN program. POSBINDU, a community-based program for hypertension screening and prevention[11], was added to the several existing NCD-related programs Indonesia. These include Prolanis (Program Pengendalian Penyakit Kronis), a community-based hypertension and diabetes management program affiliated with primary care[12] and Posyandu Lansia, a community-based NCDs screening and management for the elderly.[13] Despite these efforts, the awareness and control of hypertension are still relatively low: only 25% of people with elevated blood pressure are aware of their condition, and only 54% of people diagnosed with hypertension take routine medication.[4,14,15] These conditions are still below the "rule of halves" for hypertension management, which recommends that 50% of hypertension patients be aware of their condition, with half of whom should be treated.[16,17]

For peer review only - http://bmjopen.bmj.com/site/about/guidelines.xhtml

A process evaluation is important in assessing the implementation, to identify barriers, and provide specific recommendations for improvement of POSBINDU. Previous studies have evaluated the effectiveness of the POSBINDU implementation.[11,18] However, they were lacking on the evaluation of contextual barriers in POSBINDU implementation. This study aims to portray the implementation of POSBINDU and its contextual barriers, to provide recommendations for better hypertension and its risk factors screening, and optimal linkage to care in Indonesia.

Methods

Setting

POSBINDU is a community-based activity run by community health cadres (volunteers) and supervised by primary health care (PHC) officials. POSBINDU aims to empower communities in screening for NCDs and the risk factors, targeting individuals above 15 years old, particularly those of productive age.[19,20] The main activities include screening for NCDs (mainly hypertension and diabetes) and the risk factors (i.e., smoking, diet, physical activity, obesity). Further, POSBINDU also provides health education and facilitate referral to PHC.[19] For this study, we focus on POSBINDU implementation in screening of hypertension and its risk factor, particularly, since only 30% of hypertensive patients in Indonesia received formal diagnosis.[15]

Study Design

This was a concurrent mixed-methods study in seven districts in three provinces in Indonesia (Central Java, East Java, and North Sumatra). We purposely selected provinces with relatively high prevalence of NCDs based on a national health survey conducted in 2018.[21] Cross-sectional study by obtaining POSBINDU reports were conducted for the quantitative evaluation, whereas case study was conducted to explore barriers of POSBINDU implementation.

Ethnical approval

The study was approved by the ethical review board at Universitas Gadjah Mada, reference number KE/FK/0648/2019. The participants of Focus Group Discussion gave informed consent before participating in this study.

Data Collection

Within every one of the three provinces, we selected two districts: one city representing urban communities, and one district representing rural communities. In Central Java, an additional city was also selected (Figure 1). The rural/urban classification is based on population density and facilities available in the communities. For each district, approximately 50% of the primary health care (PHC) were selected as areas for data collection (n PHC=100). Within the PHC, we collected data for quantitative process evaluation from all active POSBINDU in the areas (n POSBINDU=475). Due to the different number of POSBINDU within each district or PHCs, the number of POSBINDU visitors as well as visits varies by the areas. In most POSBINDU, online/electronic data were not available; Hence, data on participation were manually collected from the POSBINDU register. Data from 2018-2019 were collected, except for Central Java, in which data were available through September 2019.

For the qualitative approach, 21 focus group discussions (FGDs) and 2 in-depth interviews were held among a total of 223 informants: 22 from Districts Health Department, 101 from Primary Health Care (PHC) facilities and 100 POSBINDU cadres. The two in-depth interviews were conducted with health districts department officials. Within each district, we conducted purposive sampling to recruit health officials responsible for POSBINDU program from the district's health department, and primary health care. We also recruit 2-3 cadres from each PHC based on list of cadres obtained from PHC officials. These participants were recruited to obtain information on POSBINDU implementation facilitators and barriers. The size of the FGDs was on average 10 persons (min 4, max 18). Verbatim transcripts of the

For peer review only - http://bmjopen.bmj.com/site/about/guidelines.xhtml

FGD's were made for qualitative analyses. The FGD facilitators had public health background and experience in conducting qualitative research. All facilitators attended the preparatory meeting to discuss the FGDs and interview guidelines, to obtain similar perception regarding the aims of FGDs and interviews and items of the FGD guidelines.

Insert Figure 1 here

Outcome and variables measurements

Missed opportunities in hypertension screening were quantified by the proportion of POSBINDU visitors getting the risk factors anamnesis, and measurement of anthropometric tests, blood pressure and cholesterol. Analyses was conducted on each indicator to provide more detailed information on specific components of screening which were lacking. Sociodemographic variables which were available on the POSBINDU register, were included in the analyses: sex, age, and level of education. Age was classified into several groups based the Indonesian Ministry of Health classification for age (youth = 15-24 years old, adult = 25-44 years old, pre-elderly = 45-59 years old, and elderly => 60 years old). Occupation was not included in the analyses due to high missing value in the POSBINDU reports (>60%).

Personal and family history of NCDs were also obtained, which include seven (7) diseases: hypertension, diabetes, heart disease, stroke, asthma, cancer, and high blood cholesterol. Complete personal/family history variables were coded 1 if all information was available and coded 0 if at least one of the disease histories was missing. Any personal/family history variables were coded 1 if at least one of the disease histories was available and coded 0 if all of the history information was missing.

We also generate variable "incomplete information" which represent whether the individual received the recommended procedure (history taking, anthropometric measurement, blood pressure measurement, and blood examination). The proportion presented in the analyses, described the individuals who did not receive the complete recommended procedure.

We used the logic model framework for process evaluation to assess the implementation of POSBINDU. We adopted several indicators from the current literature on the use of logic model in process evaluation of community-based health intervention [22–24]. The FGDs theme as well as indicators of the secondary data developed based on the literature, were discussed with officials from health department and PHC officials in one pilot site for finalization. We further explored the barriers of POSBINDU implementation using a qualitative approach.

For peer review only

Analyses

Statistical analyses were conducted using STATA, to calculate the proportion of activities and outcomes. We further conduct Chi-square, T-test, and ANOVA to assess the statistical significance of the differences. Analyses were conducted on missing information, reflecting whether specific procedure in POSBINDU were carried out and reported. Further analyses on proportion of hypertension and BMI status were also conducted. The two indicators were reported due to relatively high availability of these data (92% and 76%) compared to other indicators. Verbatim transcript from FGDs and in-depth interviews recordings were analyzed. Content analysis was applied for the qualitative data to ascertain barriers for the POSBINDU implementation in Indonesia by two independent researchers. To enhance trustworthiness, we assess barriers of POSBINDU from several sources for triangulation purposes: health and PHC officials to reflect implementer's perspective, and cadres to reflect implementers and users' perspective. During data analyses, we also discuss the findings with representative of the FGD participants, i.e., member checking. Parallel analyses were conducted to synthesize the findings from the quantitative and qualitative approaches. Weaving

For peer review only - http://bmjopen.bmj.com/site/about/guidelines.xhtml

technique, analyzing the quantitative and qualitative findings together by theme or concept, was used to integrate the findings.[25]

Patients and public involvement

Patients or the public were not directly involved in the design, or conduct, or reporting, or dissemination plans of our research.

Results

Participation of community for hypertension screening in POSBINDU

Data from 114,581 POSBINDU visits (54,224 participants) were analyzed. The findings showed similar patterns in the districts and provinces: more female and elderly participants. Approximately 80% were female participants, with the highest proportion of female participants in rural North Sumatra (95.5%). Meanwhile, in Java, a higher proportion of female participants were observed in urban areas (Table 1).

Table 1. Characteristics of POSBINDU Participants within the Three Provinces in Indonesia (POSBINDU Register, 2018-2019)

Characteristics	North Sumatra		East Java		Central Java		
	Rural	Urban	Rural	Urban	Rural	Urban	Total
Number of individuals	5,103	10,999	23,053	4,983	3,398	6,688	54,224
Number of PHC	11	23	29	9	11	17	100
Number of POSBINDU	38	38	283	27	27	62	475
Categorical (%, SE)							
Female	95.5 (0.3)	71.3 (0.4)	76.2 (0.3)	86.7 (0.5)	73.7 (0.8)	88.2 (0.4)	79.4 (0.2)
Age							
15-24	8.7 (0.4)	6.1 (0.3)	6.2 (0.2)	7.8 (0.4)	13.3 (0.7)	3.8 (0.3)	6.7 (0.1)
25-44	38.1 (0.8)	24.9 (0.5)	33.0 (0.3)	48.7 (0.8)	47.7 (1.0)	28.9 (0.7)	22.2 (0.2)
45-59	30.7 (0.7)	37.6 (0.6)	33.2 (0.3)	31.0 (0.6)	30.1 (0.9)	42.4 (0.7)	24.8 (0.2)
>60	22.4 (0.6)	31.3 (0.6)	27.6 (0.3)	12.5 (0.5)	8.9 (0.6)	24.9 (0.6)	24.7 (0.2)
Education**							
PS	2.6 (0.2)	15.3 (0.3)	57.3 (0.3)	50.5 (0.7)	41.1 (0.8)	25.2 (0.5)	38.0 (0.2)
HS	0.3 (0.07)	0.7 (0.1)	0.3 (0.0)	1.3 (0.2)	2.3 (0.3)	1.8 (0.2)	0.8 (0.0)
Univ	0.0 (0.0)	2.7 (0.2)	0.4 (0.04)	4.9 (0.3)	4.9 (0.4)	2.1 (0.2)	1.8 (0.1)
Missing	97.1 (0.2)	81.4 (0.4)	41.9 (0.3)	43.3 (0.7)	51.8 (0.9)	70.8 (0.6)	59.4 (0.2)
Number of visits							
1 time	87.0 (0.4)	77.4 (0.3)	68.4 (0.3)	65.6 (0.7)	84.5 (0.6)	56.4 (0.6)	71.2 (0.1)
2-6 times	12.9 (0.5)	21.7 (0.3)	21.1 (0.2)	23.9 (0.6)	13.3 (0.6)	35.1 (0.6)	22.0 (0.1)
7-12 times	0.1 (0.0)	0.6 (0.1)	6.3 (0.2)	6.0 (0.3)	2.1 (0.2)	5.9 (0.3)	5.3 (0.1)
>12 times	0 (0.0)	0.2 (0.1)	4.1 (0.1)	4.5 (0.3)	0.1 (0.0)	2.6 (0.2)	1.4 (0.1)
Continuous (mean, SE)							
Age	46.4 (0.2)	51.7 (0.2)	49.7 (0.1)	43.4 (0.2)	41.0 (0.3)	50.4 (0.2)	48.6 (0.8)
Number of visits	1.2 (0.8)	1.4 (0.1)	2.5 (0.2)	2.7 (0.5)	1.4 (0.2)	2.5 (0.3)	2.1 (0.1)

Notes:
Within province, rural-urban comparisons are significant at 0.05

For peer review only - http://bmjopen.bmj.com/site/about/guidelines.xhtml

Between provinces comparisons are significant at 0.05
Differences in proportion tested using Chi squares
SE= standard error
**Education = HS (High school) PS (Primary school/less) Univ (University/college), Missing = data missing

Despite the relatively high missing information on age (n missing= 12,084, or 22.3%), we found that the participants were on average of older age, with roughly 50% of participants aged over 50 years old (Table 1). The highest proportion of participants >60 years old were observed in rural East Java (31.3%), with mean age of 51.7 years old. We measured the youngest POSBINDU participants in rural Central Java (mean age 41.0 years old). Meanwhile, the missing information on education level were higher (almost 60%), with even higher proportion in North Sumatra.

In the span of the two years of secondary data collection, we found that, on average, the participants visit POSBINDU twice, with the lowest average of visits in North Sumatra (rounded to 1 visit/participant). Approximately 38,628 (71.2%) of participants visit POSBINDU once for two years, and 761 (1.4%) visits POSBINDU more than 12 times.

Table 2. Missed Opportunity in Hypertension Screening and Risk Factors Characteristics within POSBINDU Participants (POSBINDU Register, 2018-2019)

Characteristics	North Sumatra		East Java		Central Java		
	Rural %(SE)	Urban %(SE)	Rural %(SE)	Urban %(SE)	Rural %(SE)	Urban %(SE)	Total %(SE)
Missing Information in All Visits							
n	6,061	15,774	57,504	13,422	4,925	16,895	114,581
Personal history (complete)	99.3 (0.1)	92.6 (0.2)	67.9 (0.2)	42.5 (0.4)	62.1 (0.7)	95.2 (0.2)	73.8 (0.1)
Family history (complete)	99.4 (0.1)	92.6 (0.2)	65.1 (0.2)	39.7 (0.4)	61.6 (0.7)	95.9 (0.2)	72.1 (0.1)
Personal history (any)	88.2 (0.4)	88.5 (0.3)	56.4 (0.2)	17.2 (0.3)	54.2 (0.7)	91.0 (0.2)	62.9 (0.1)
Family history (any)	97.5 (0.2)	88.9 (0.3)	57.3 (0.2)	28.9 (0.4)	53.5 (0.7)	93.8 (0.2)	65.7 (0.1)
Height	42.7 (0.6)	19.0 (0.2)	15.6 (0.2)	20.7 (0.3)	30.6 (0.7)	23.3 (0.3)	19.9 (0.1)
Weight measurement	35.8 (0.6)	16.0 (0.2)	18.8 (0.2)	5.6 (0.2)	8.9 (0.4)	12.3 (0.3)	16.4 (0.1)
Waist circumference	49.5 (0.6)	36.0 (0.3)	15.6 (0.2)	12.7 (0.2)	36.9 (0.6)	63.6 (0.4)	27.8 ().1)
Blood pressure	1.8 (0.2)	5.8 (0.2)	9.8 (0.1)	4.6 (0.2)	4.2 (0.3)	6.3 (0.2)	7.4 (0.1)
Blood cholesterol	87.0 (0.4)	80.1 (0.3)	81.9 (0.2)	97.4 (0.1)	91.1 (0.4)	82.0 (0.3)	84.2 (0.1)
Incomplete information	99.6 (0.1)	95.1 (0.2)	98.1 (0.1)	99.6 (0.1)	99.6 (0.1)	99.6 (0.1)	98.2 (0.1)
**Missing Information in First Visits**							
n	5,103	10,999	23,053	4,983	2,298	6,688	54,224
Personal history (complete)	99.2 (0.1)	89.9 (0.3)	72.8 (0.3)	35.1 (0.7)	49.3 (0.9)	92.6 (0.3)	76.3 (0.2)
Family history (complete)	99.3 (0.1)	89.8 (0.3)	71.3 (0.3)	41.4 (0.7)	50.9 (0.9)	93.5 (0.3)	76.4 (0.2)
Personal history (any)	88.2 (0.5)	85.1 (0.3)	58.3 (0.3)	15.3 (0.5)	40.1 (0.8)	86.9 (0.4)	68.3 (0.2)
Family history (any)	97.3 ().2)	85.2 (0.3)	60.6 (0.3)	30.0 (0.6)	40.1 (0.8)	90.5 (0.4)	65.0 (0.2)
Height	41.8 (0.7)	19.0 (0.4)	14.8 (0.2)	21.1 (0.6)	20.4 (0.7)	23.0 (0.5)	20.1 (0.2)

For peer review only

For peer review only - http://bmjopen.bmj.com/site/about/guidelines.xhtml

Weight	35.3 (0.3)	15.0 (0.3)	16.7 (0.2)	47.6 (0.3)	7.2 ().4)	12.9 (0.4)	15.9 (0.1)
Waist circumference	48.9 ().7)	33.6 (0.4)	11.1 (0.2)	12.9 (0.5)	23.0 ().7)	66.1 (0.6)	26.9 (0.2)
Blood pressure	1.7 (0.2)	5.4 (0.2)	4.9 (0.1)	5.0 (0.3)	3.2 (0.3)	7.0 (0.3)	4.9 (0.1)
Blood cholesterol	86.1 (0.5)	75.7 (0.4)	76.6 (0.3)	97.9 (0.2)	92.3 (0.4)	79.1 (0.5)	80.6 (0.2)
Incomplete information	99.6 (0.1)	93.0 (0.2)	96.7 (0.1)	99.7 (0.1)	99.4 (0.1)	99.4 (0.1)	97.01 (0.1)
**Risk Factors Screening in All Visits**							
**n**	3,423	12,015	45,108	10,484	3,374	12,750	87,154
BMI							
Normal	48.3 (0.9)	48.2 (0.5)	51.3 (0.2)	44.6 (0.5)	52.3 (0.9)	46.4 (0.4)	49.3 (0.2)
Underweight	4.9 (0.4)	4.5 (0.2)	8.0 (0.1)	4.5 (0.2)	9.1 (0.5)	3.8 (0.2)	6.3 (0.1)
Overweight	31.9 (0.8)	34.4 (0.4)	30.8 (0.2)	34.7 (0.4)	29.1 (0.8)	34.2 (0.4)	32.3 (0.2)
Obese	14.9 (0.6)	12.9 (0.3)	10.0 (0.3)	16.3 (0.4)	9.4 (0.5)	15.7 (0.3)	12.1 (0.1)
n	5,942	14,835	51,784	12,773	4,717	15,814	105,865
Hypertension	35.4 (0.6)	28.0 (0.4)	42.5 (0.2)	33.7 (0.4)	25.6 (0.6)	35.9 (0.4)	37.2 (0.1)
**Risk Factors Screening in First Visits**							
**n**	2,925	8,440	18,820	3,850	2,678	5,078	41,791
BMI							
Normal	49.3 (0.9)	48.0 (0.5)	51.5 (0.4)	45.3 (0.8)	52.4 (1.0)	44.4 (0.7)	49.3 (0.2)
Underweight	4.8 (0.4)	4.5 (0.2)	7.7 (0.2)	5.1 (0.4)	10.1 (0.6)	4.2 (0.3)	6.3 (0.1)
Overweight	32.0 (0.9)	34.2 (0.5)	30.4 (0.3)	33.3 (0.8)	28.7 (0.9)	35.1 (0.7)	32.0 (0.2)
Obese	13.9 (0.6)	13.3 (0.4)	10.4 (0.2)	16.3 (0.6)	8.8 (0.5)	16.3 (0.5)	12.4 (0.2)
n	5,008	10,379	21,858	4,725	3,288	6,201	51,459
Hypertension	34.5 (0.7)	28.5 (0.4)	40.5 (0.3)	31.6 (0.7)	25.1 (0.8)	37.9 (0.6)	35.3 (0.2)

Notes:
Within province, rural-urban comparisons are significant at 0.05
Between provinces comparisons are significant at 0.05
Differences in proportion tested using Chi squares
SE= standard error

We further observed the relatively high missing information for hypertension screening across the districts, with the following general pattern. First, a relatively high proportion of missing information concerning the personal and family history, with East Java having the lowest proportion. Second, a relatively lower proportion of missing data on anthropometric measurements (less than 50%). Third, we found the highest proportion of available data for blood pressure measurements in all the seven districts. Last, our analysis identified higher missing values for blood cholesterol measurements (84,2%). For all measurements, there were significant differences between the three provinces, as well as between the rural and urban areas within the provinces (Table 2).

Based on available data, we found that obesity seems to be more prevalent in urban areas in Java, but relatively similar in North Sumatra. In contrast, hypertension was more prevalent in a rural area for East Java and North Sumatra but was more common in urban districts of Central Java (Table 2). However, these data should be interpreted cautiously due to the relatively high missing data on the measurements.

For peer review only - http://bmjopen.bmj.com/site/about/guidelines.xhtml

Barriers for the screening of hypertension in POSBINDU

The qualitative data supported the quantitative finding about lacking participation of male and younger population to POSBINDU. In the FGDs, cadres and health officials stated the barriers for male and younger participants to attend POSBINDU, including the inconvenience of POSBINDU schedule, as well as low awareness for hypertension screening (Table 3).

Table 3. Qualitative analyses of Focus Group Discussion amongst POSBINDU Cadres, Primary Health Care and Health Department Officials

Themes	Category	Codes
Suboptimal target population and gap in policy	Participants' characteristics	Younger adults rarely participate
		Lack of male participants
	Barrier to participations	Schedule incompatibility
		Low awareness for screening
		Lack of role model for screening
	Ineffective policy and coordination	Lack of prioritization for NCD
		Implementation gap of national policy/program at the local level
		The need for coordination with different stakeholders
		The need for coordination among NCD-related programs
Lack of human resources in terms of capability and quantity for hypertension screening	Cadres have multiple tasks, with time constraints	POSBINDU cadres often have to multitask and handling other community programs
		Cadres are volunteers with other obligations
	Cadres' competencies	Lack of knowledge on hypertension and other NCD
		Lack of ability to conduct measurements and provide health education
		Lack of ability to conduct recording and reporting
	Lack of NCD program officers for supervision and reporting	Lack of NCD program officers at PHC
		Most program officers are responsible for multiple tasks/programs
		Lack of reporting officers
	Provision of Referral Counselling	The participant with hypertension is not always referred to PHC
		Lack of counseling to participants before the referral made
		POSBINDU has referral form, but rarely used
		Treatment for the referral is covered by their health insurance
Lack of resources for hypertension screening and prevention	Equipment for hypertension screening	The equipment is sometimes incomplete
		Equipment maintenance is inadequate
		Limited logistics for cholesterol measurement
	Lack of budget	POSBINDU is funded by the government, stakeholder (private sectors) or community
		Lack of budget for POSBINDU activities

For peer review only - http://bmjopen.bmj.com/site/about/guidelines.xhtml

		Lack of budget for cadres training and incentives
	Health education material	Lack of health education materials
	Infrastructure for recording and reporting	Not all cadres have laptops
		Limited internet connection in some areas
		Most POSBINDU stations use manual reporting
Time constraints for implementation based on MOH standard	The complexity of activities and time limitation	The time required for examination is too long
		Too many information needs to be asked and filled out
		The referral form is rarely used
	The complexity of reporting forms	Many forms need to be filled, while time is limited
		A simplified form in checklist format is preferred

The need for role model from community leader to improve participation and the barrier for participation, particularly among males is highlighted by these quotes:

" Yes, we don't have a lot of men (participants), because they are working " (Cadre, FGD#21)

" In our POSBINDU, *the awareness for early screening is still low. Only several people come (to* POSBINDU*), younger people don't want to come because* (POSBINDU *is conducted) during working days " (Cadre, FGD#3)*

" ...Socialization for this (POSBINDU) is needed, often, the community leader in our area don't want to participate because they are afraid to be screened" (Cadre, FGD#2)

".. when I asked the communities, why they did not come to POSBINDU, or why there were only few people, they said because I (the community member) were not sick, so why do I need to get (health) check-up (?). So, they were not aware that POSBINDU is not only for those who are sick" (Health official, FGD#19)

" I asked POSBINDU (participant), why elderly? Where are the younger population? And they said that the young stayed at home because they were embarrassed if they have diseases.. " (Health official, FGD#16)

Lack of priority and overlap of NCD-related programs also contribute to a suboptimal target population of POSBINDU, as illustrated by the following quote:

" (*NCD) is not a priority program, hence, there's a lack of commitment between the superior (health department) with the program officials, for example." (Health official, FGD#7)*

"...(different department in) the Ministry of Health focus on specific diseases, such as diabetes and cancer... However, in the community, (the programs) become general. (We) run Polindes, (Posyandu) Lansia, POSBINDU, School Health Program. In my opinion, the regulation is rigid and detailed, but the implementation is mixed (overlap). If we want to give optimum results, it takes efforts." (Health official, FGD#10).

Several barriers for implementation were revealed. The cadres and health officers often have to run several different programs. The FGDs also revealed a lack of capability of cadres to conduct measurements for hypertension screening, providing health education, and also conducting the recording and reporting of the POSBINDU activities and measurements. The informants also

For peer review only

mentioned a lack of resources, including budget, equipment, and logistics to conduct all the measurements.

> *" One person can hold 5 positions in PHC activities… POSBINDU cadres, Posyandu Lansia cadres, and other programs.. " (Cadre, FGD#11)*

> *" (cadres of) Posbindu do not have laptop nor cell phone for the reporting application (of* POSBINDU*), hence, we report to PHC manually" (Cadre, FGD#14)*

The barriers also include the complexities of the activities and measurements, as well as extensive reporting forms, which require a long time to be completed.

> *" …it takes a long time, because of the measurements and stages (of* POSBINDU *activities) " (PHC officer, FGD#8)*

> *" …*POSBINDU *report is too time-consuming, because it is long (detail), including identity, cell phone number, address, and others… and it has to be filled out every month." (PHC officer, FGD#6)*

Interestingly, in several districts, we found the implementation of mobile POSBINDU, moving from one community to the other within the same subdistricts.

> *"Our* POSBINDU *is mobile, we have ten communities, so every week, we move from one community to the next, focusing on people 15-59 years old." (Cadre, FGD#18)*

We further synthesized the quantitative and qualitative results. We categorized the barriers into three main parts: 1) input, reflecting the target population/coverage of POSBINDU, 2) process, describing the implementation of POSBINDU activities, and 3) output, reflecting the recording and reporting process of POSBINDU (Figure 2). Results show that in both approaches we found lacking participation of male and younger people in POSBINDU. Lack of priority for NCD screening and ineffective coordination among stakeholders, combined with lack of awareness and access might attribute to this finding. The high missed opportunity, particularly in history taking and measurements, were likely due to the complexity of the activities/measurements, as well as lack of resources. The high missing data also stem from the complexity of the forms and lack of capability for online reporting.

Insert Figure 2 here

Discussion

In this study, we revealed missed opportunities in input, activities, and output of POSBINDU implementation in screening for hypertension and its risk factors. Several contextual barriers were identified. The suboptimal coverage was possibly due to lack of priority for NCD screening, lack of awareness and access, and overlap of NCD-related program. The suboptimal activities and reporting were likely caused by a lack of resources, as well as limited time to perform the complexities of activities and reporting according to MOH guideline.

The missed opportunity to screen male and younger population that we found in this study is particularly concerning. Although the prevalence is lower than of the older population, hypertension prevalence among young Indonesian is still relatively high (28%).[26] While the target population of POSBINDU is listed as those 15 years or older, the elderly are usually targeted in Posyandu Lansia, a community-based screening and management for the elderly population.[27] Awareness is also lower in male and younger adults, signaling the need to screen this population.[3] Ideally, POSBINDU becomes the "gatekeeper" for screening in the community. Hypertensive and diabetic patients were

For peer review only - http://bmjopen.bmj.com/site/about/guidelines.xhtml

For peer review only

then referred to PHC and joined Prolanis, a community-based activity funded by the health insurance program, for management of chronic diseases patients.[12]

Furthermore, with a lack of male participation, POSBINDU is missing one of the key target populations for risk factors screening: smokers. Analysis of a national survey in 2014 reported 32% prevalence of smoking, with approximately 40% of males aged 15-55 years old and 14% of male adolescents are current smokers.[28–30] Further, 20% of Indonesia's total chronic diseases are attributed to smoking, with hypertension as the highest proportion.[31] Screening for hypertension and its risk factors earlier, combined with lifestyle-based interventions effectively avoid future complications.[32,33]

We also revealed the need to prioritize and reorganize the current NCD-related programs, to address the suboptimal coverage and the overlap. An example of the gap between the national recommendation and local implementation is reflected in the coordination of existing NCD-related programs: POSBINDU, Posyandu Lansia, and PANDU PTM. In the MOH, the PANDU PTM (Pelayanan Terpadu Penyakit Tidak Menular, Integrated Health Services for NCDs) and POSBINDU are regulated under the Directorate for Disease Management, while Posyandu Lansia is under the Directorate of Public Health. Despite the different directorates, the implementation at community level is often conducted simultaneously and often overlap. Reporting, however, is conducted separately. Hence, as previous studies have noted, we also recommend the need of comprehensive and coordinated NCDs prevention program in Indonesia.[34–36]

The relatively high missed opportunity in screening for hypertension risk factors, as well as sociodemographic characteristics found in this study, portrays suboptimal implementation of POSBINDU. This can be caused by a lack of recording and reporting (monitoring and evaluation fidelity) or lack of measurement (implementation fidelity). In our further elaboration during the FGDs, we found that lack of human resources might contribute to the suboptimal implementation of POSBINDU. Our findings revealed the need to train cadres to improve their skills and efficiency in conducting the measurements and history taking, as well as reporting the measurements. This is in line with findings from Meinema et al (2017 and Abdell-All et al (2018)[37,38]. Our findings also imply the complexities of the activities and reporting of POSBINDU which lead to ineffective implementation. It is important to ensure that valuable screening information can be recorded and followed up, for better intervention. A simplified screening program with integrated reporting is needed.

In this study, we also discovered lack of financial resources and equipment as barriers to POSBINDU implementation. The integration of POSBINDU and PANDU PTM to the national health insurance scheme might be important to ensure the sustainability of funding for the program. Integration of POSBINDU into the national health insurance can also improve participation of the working population, most of whom are covered by the national health insurance.[39] Previous studies have reported an increase in uptake of service by health insurance membership.[40–42]

Based on our findings, we identified two main areas that needs to be improved: coverage and implementation of POSBINDU. To improve coverage of POSBINDU, there are two important steps that we recommend. First, an integrated approach with collaboration amongst different programs and directorates to reduce the overlap and simplify the POSBINDU implementation at the PHC and community level. PANDU PTM as the adaptation of WHO PEN,[43] needs to be implemented in a wider scale. Second, redirecting the target population of hypertension screening, to cover also younger and male population. A workplace-based screening program which can address the barriers identified in the qualitative findings is recommended.[44,45] For this younger population, the use of mobile technology for monitoring of risk factors and measurement might be effective. Previous

For peer review only - http://bmjopen.bmj.com/site/about/guidelines.xhtml

studies have reported the effectiveness of mobile health for hypertension screening and risk stratification.[46,47]

To improve the implementation and components of POSBINDU activities, a simplified algorithm to screen and refer the target population is needed. The algorithm needs to be developed both in the electronic format and manual format to address the different capabilities of community cadres and resources in the community. Simplifying the program and reporting systems will also reduce the workload of PHC and district health officials. Further, a clear algorithm for the referral of "screened" cases to PHC is important. The readiness of the PHCs also needs to be improved to adequately manage the potential surge in referred cases. Lastly, there is a need to integrate hypertension and CVD screening program into the national health insurance system. Hence, ensuring the sustainability of funding and resources of the program. With these approaches, comprehensive screening for hypertension and CVD along the continuum of care might be more effective.

This study has several limitations. First, the proportion of our measures are not reflective for the whole target population of POSBINDU, since the participants were mostly female and of older age. The characteristics of our sample, which are generally older with a higher proportion of females, drive the proportion higher than that of the general population in Indonesia. However, this study reflects the current participants of POSBINDU. Second, we used a secondary data collection by POSBINDU cadres, the high number of missing data that we presented in this study, probably stem from two main sources: omissions in reporting or a true lack in measurement/activities. Nevertheless, both the activities and reporting are important in NCDs screening, particularly in the follow-up. The secondary data also prone to measurement bias, particularly, with the variations in POSBINDU measurements by cadres. The Ministry of Health provided guidelines in the measurement for hypertension in POSBINDU, however, the implementation might vary. The high missing information on several sociodemographic characteristics i.e., occupation and education, also limit our ability to conduct multivariable analyses. Another limitation of this study is we have not included the perspective of POSBINDU participants in the FGDs. Instead, we considered the POSBINDU cadres to represents the voice of both the implementers as well as users. However, we include the perspective of the POSBINDU participants in the baseline of our prospective data collection (ongoing).

Despite the limitation, there are several strengths of the study: First, to our knowledge, this was the first relatively large evaluation of POSBINDU. Second, the use of a mixed-methods study design, and therefore, providing more comprehensive information on POSBINDU implementation. Third, the study also investigates the contextual factors that should be addressed in the improvement of the community-based hypertension screening program in Indonesia. This study might provide insights into POSBINDU implementation in other areas in Indonesia, and can be the basis for further recommendation to improve POSBINDU implementation.

Conclusion

This study showed the suboptimal implementation of POSBINDU activities. Particularly, the missed opportunity in screening for hypertension risk factors in Indonesia. The barriers include a lack priority for NCDs, lack of awareness and access for subpopulation, and several implementation barriers: capability, resources, and protocols. An innovative approach to simplify and improve the capacity of POSBINDU is in preparation to optimize the screening and linkage to hypertension care in Indonesia. This study provides evidence-based recommendations in improving the current implementation of POSBINDU, in the Indonesian context.

For peer review only - http://bmjopen.bmj.com/site/about/guidelines.xhtml

Data Statement

The de-identified data from this study is available upon request to the corresponding or first author pending thorough review of request and adherence to the Indonesian government regulation on data sharing.

Author Contributions

VW, AP, RFP, EP, YM, JK and JLD contributed in the design of the study. VW, A, RFP, EP, YM, B, and M participate actively in study implementation, including in data collection. VW, YM, S, and RFP analyzed the quantitative data. AP, EP, and B analyzed the qualitative data. VW, AP, RFP, S, and YM draft the manuscript with all co-authors revised critically. All authors read and approved the final manuscript.

Funding

This work was supported by the Horizon 2020 research program of the European Union. This study was co-financed by the Horizon 2020 research program of the European Union, under grant 825026 Scaling Up NCDs interventions in South East Asia.

Conflict of Interest

None declared

License

The Corresponding Author has the right to grant on behalf of all authors and does grant on behalf of all authors, an exclusive licence (or non exclusive for government employees) on a worldwide basis to the BMJ Publishing Group Ltd ("BMJ"), and its Licencees to permit this article (if accepted) to be published in The BMJ's editions and any other BMJ products and to exploit all subsidiary rights, as set out in our licence.

Acknowledgment

Figure 1. Study Sample Selection

Figure 2. Synthesis of the Quantitative and Qualitative Findings

References

1 Basu S, Millett C. Social epidemiology of hypertension in middle-income countries: Determinants of prevalence, diagnosis, treatment, and control in the WHO SAGE study. *Hypertension* 2013;**62**:18–26. doi:10.1161/HYPERTENSIONAHA.113.01374

2 Mills KT, Bundy JD, Kelly TN, *et al.* Global disparities of hypertension prevalence and control. *Circulation* 2016;**134**:441–50. doi:10.1161/CIRCULATIONAHA.115.018912

3 Hussain MA, Al Mamun A, Reid C, *et al.* Prevalence, awareness, treatment and control of hypertension in Indonesian adults aged ≥40 years: Findings from the Indonesia Family Life Survey (IFLS). *PLoS One* 2016;**11**:1–16. doi:10.1371/journal.pone.0160922

4 Peltzer K, Pengpid S. The Prevalence and Social Determinants of Hypertension among Adults in Indonesia: A Cross-Sectional Population-Based National Survey. *Int J Hypertens* 2018;**2018**. doi:10.1155/2018/5610725

5 Purnamasari D. The Emergence of Non-communicable Disease in Indonesia. *Acta Med Indones* 2018;**50**:273–4.http://www.ncbi.nlm.nih.gov/pubmed/30630990

6 Vos T, Abajobir AA, Abbafati C, *et al.* Global, regional, and national incidence, prevalence, and

For peer review only - http://bmjopen.bmj.com/site/about/guidelines.xhtml

years lived with disability for 328 diseases and injuries for 195 countries, 1990-2016: A systematic analysis for the Global Burden of Disease Study 2016. *Lancet* 2017;**390**:1211–59. doi:10.1016/S0140-6736(17)32154-2

7 Gaziano T. Prevention and treatment of chronic diseases in developing countries. 2011;:1–20.http://www.un.org/esa/population/publications/expertpapers/2011-2-gaziano.pdf

8 Allen L, Williams J, Townsend N, *et al.* Socioeconomic status and non-communicable disease behavioural risk factors in low-income and lower-middle-income countries: a systematic review. *Lancet Glob Heal* 2017;**5**:e277–89. doi:10.1016/S2214-109X(17)30058-X

9 Rosengren A, Smyth A, Rangarajan S, *et al.* Socioeconomic status and risk of cardiovascular disease in 20 low-income, middle-income, and high-income countries: the Prospective Urban Rural Epidemiologic (PURE) study. *Lancet Glob Heal* 2019;**7**:e748–60. doi:10.1016/S2214-109X(19)30045-2

10 WHO. Package of Essential Noncommunicable (PEN) Disease Interventions for Primary Health Care. *Geneva World Heal Organ* 2010;:1–66.papers2://publication/uuid/FC98856B-4122-4325-826F-20FA6B89DEBD

11 Ferdianto A, Tamtomo DG, Sulaeman ES. Does the Integrated Health Post have Contextual Effect on Tertiary Preventive Behavior among Hypertensive Patients? A Multilevel Analysis Evidence from Surakarta. *J Heal Promot Behav* 2019;**4**:224–34. doi:10.26911/thejhpb.2019.04.03.07

12 Khoe LC, Wangge G, Soewondo P, *et al.* The implementation of community-based diabetes and hypertension management care program in Indonesia. *PLoS One* 2020;**15**:1–9. doi:10.1371/journal.pone.0227806

13 Yamada M, Hapsari ED, Matsuo H. Behaviors toward noncommunicable diseases prevention and their relationship with physical health status among community-dwelling, middleaged and older women in Indonesia. *Int J Environ Res Public Health* 2020;**17**. doi:10.3390/ijerph17072332

14 Maharani A, Sujarwoto, Praveen D, *et al.* Cardiovascular disease risk factor prevalence and estimated 10-year cardiovascular risk scores in Indonesia: The SMARThealth Extend study. *PLoS One* 2019;**14**:1–13. doi:10.1371/journal.pone.0215219

15 Turana Y, Tengkawan J, Soenarta AA. Asian management of hypertension: Current status, home blood pressure, and specific concerns in Indonesia. *J Clin Hypertens* 2020;**22**:483–5. doi:10.1111/jch.13681

16 Scheltens T, Bots ML, Numans ME, *et al.* Awareness, treatment and control of hypertension: The 'rule of halves' in an era of risk-based treatment of hypertension. *J Hum Hypertens* 2007;**21**:99–106. doi:10.1038/sj.jhh.1002123

17 Chen R, Tunstall-Pedoe H, Morrison C, *et al.* Trends and social factors in blood pressure control in Scottish MONICA surveys 1986-1995: The rule of halves revisited. *J Hum Hypertens* 2003;**17**:751–9. doi:10.1038/sj.jhh.1001612

18 Alfiyah A, Pujiyanto P. an Analysis on the Implementation of the Integrated Guidance Post (Posbindu) Activities for Non-Communicable Diseases At Bogor City in 2018. *J Indones Heal Policy Adm* 2019;**4**:11–5. doi:10.7454/ihpa.v4i1.2388

19 Ministry of Health (MOH) - Kementerian Kesehatan. Petunjuk Teknis Pos Pembinaan Terpadu Penyakit Tidak Menular (Posbindu PTM). 2012. http://p2ptm.kemkes.go.id/uploads/2016/10/Petunjuk-Teknis-Pos-Pembinaan-Terpadu-

For peer review only

For peer review only - http://bmjopen.bmj.com/site/about/guidelines.xhtml

Penyakit-Tidak-Menular-POSBINDU-PTM-2013.pdf

20 Moeloek NF. Indonesia national health policy in the transition of disease burden and health insurance coverage. *Med J Indones* 2017;**26**:3–6. doi:10.13181/mji.v26i1.1975

21 Ministry of Health (MOH) - Kementerian Kesehatan. Riskesdas Main Results 2018. 2018. https://www.litbang.kemkes.go.id/hasil-utama-riskesdas-2018/

22 Smith JD, Li DH, Rafferty MR. The implementation research logic model: A method for planning, executing, reporting, and synthesizing implementation projects. *medRxiv* 2020;:1–12. doi:10.1101/2020.04.05.20054379

23 Sharma S, Adetoro OO, Vidler M, *et al.* A process evaluation plan for assessing a complex community-based maternal health intervention in Ogun State, Nigeria. *BMC Health Serv Res* 2017;**17**:1–10. doi:10.1186/s12913-017-2124-4

24 Wong ST, Yin D, Bhattacharyya O, *et al.* Developing a performance measurement framework and indicators for community health service facilities in urban China. *BMC Fam Pract* 2010;**11**. doi:10.1186/1471-2296-11-91

25 Fetters MD, Curry LA, Creswell JW. Achieving integration in mixed methods designs - Principles and practices. *Health Serv Res* 2013;**48**:2134–56. doi:10.1111/1475-6773.12117

26 Sudharsanan N, Geldsetzer P. Impact of coming demographic changes on the number of adults in need of care for hypertension in Brazil, China, India, Indonesia, Mexico, and South Africa: A modeling study. *Hypertension* 2019;**73**:770–6. doi:10.1161/HYPERTENSIONAHA.118.12337

27 Rahmawati R, Bajorek B V. Access to medicines for hypertension: A survey in rural Yogyakarta province, Indonesia. *Rural Remote Health* 2018;**18**. doi:10.22605/RRH4393

28 Amalia B, Cadogan SL, Prabandari YS, *et al.* Socio-demographic inequalities in cigarette smoking in Indonesia, 2007 to 2014. *Prev Med (Baltim)* 2019;**123**:27–33. doi:10.1016/j.ypmed.2019.02.025

29 Sujarwoto S. Sleep Disturbance in Indonesia: How Much Does Smoking Contribute? *Behav Sleep Med* 2019;**00**:1–14. doi:10.1080/15402002.2019.1682584

30 Kusumawardani N, Tarigan I, Suparmi, *et al.* Socio-economic, demographic and geographic correlates of cigarette smoking among Indonesian adolescents: results from the 2013 Indonesian Basic Health Research (RISKESDAS) survey. *Glob Health Action* 2018;**11**. doi:10.1080/16549716.2018.1467605

31 Kristina SA, Endarti D, Wiedyaningsih C, *et al.* Health Care Cost of Noncommunicable Diseases Related to Smoking in Indonesia, 2015. *Asia-Pacific J Public Heal* 2018;**30**:29–35. doi:10.1177/1010539517751311

32 Saif-Ur-Rahman KM, Hasan M, Hossain S, *et al.* Non-pharmacological interventions for the prevention of hypertension in low-income and middle-income countries: Protocol for a systematic review and meta-analysis. *BMJ Open* 2018;**8**:6–11. doi:10.1136/bmjopen-2017-020724

33 Pantell MS, Prather AA, Downing JM, *et al.* Association of Social and Behavioral Risk Factors With Earlier Onset of Adult Hypertension and Diabetes. *JAMA Netw open* 2019;**2**:e193933. doi:10.1001/jamanetworkopen.2019.3933

34 Kusuma D, Kusumawardani N, Ahsan A, *et al.* On the verge of a chronic disease epidemic: Comprehensive policies and actions are needed in Indonesia. *Int Health* 2019;**11**:422–4.

For peer review only

For peer review only - http://bmjopen.bmj.com/site/about/guidelines.xhtml

doi:10.1093/inthealth/ihz025

35 Mboi N, Murty Surbakti I, Trihandini I, *et al.* On the road to universal health care in Indonesia, 1990–2016: a systematic analysis for the Global Burden of Disease Study 2016. *Lancet* 2018;**392**:581–91. doi:10.1016/S0140-6736(18)30595-6

36 Jayanna K, Swaroop N, Kar A, *et al.* Designing a comprehensive Non-Communicable Diseases (NCD) programme for hypertension and diabetes at primary health care level: Evidence and experience from urban Karnataka, South India. *BMC Public Health* 2019;**19**:1–12. doi:10.1186/s12889-019-6735-z

37 Meinema JG, Haafkens JA, Jaarsma DADC, *et al.* Development and evaluation of a culturally appropriate hypertension education (CAHE) training program for health care providers. *PLoS One* 2017;**12**:1–13. doi:10.1371/journal.pone.0178468

38 Abdel-All M, Thrift AG, Riddell M, *et al.* Evaluation of a training program of hypertension for accredited social health activists (ASHA) in rural India. *BMC Health Serv Res* 2018;**18**:1–11. doi:10.1186/s12913-018-3140-8

39 Ng JYS, Ramadani RV, Hendrawan D, *et al.* National Health Insurance Databases in Indonesia, Vietnam and the Philippines. *PharmacoEconomics - Open* 2019;**3**:517–26. doi:10.1007/s41669-019-0127-2

40 Erlangga D, Ali S, Bloor K. The impact of public health insurance on healthcare utilisation in Indonesia: evidence from panel data. *Int J Public Health* 2019;**64**:603–13. doi:10.1007/s00038-019-01215-2

41 Sharma M, Teerawattananon Y, Luz A, *et al.* Institutionalizing Evidence-Informed Priority Setting for Universal Health Coverage: Lessons From Indonesia. *Inq (United States)* 2020;**57**. doi:10.1177/0046958020924920

42 Vidyattama Y, Miranti R, Resosudarmo BP. The Role of Health Insurance Membership in Health Service Utilisation in Indonesia. *Bull Indones Econ Stud* 2014;**50**:393–413. doi:10.1080/00074918.2014.980380

43 Ministry of Health (MOH) - Kementerian Kesehatan. Pandu PTM A Community on intervention model and control's of NCD's. 2017.

44 Wang Z, Wang X, Shen Y, *et al.* Effect of a Workplace-Based Multicomponent Intervention on Hypertension Control: A Randomized Clinical Trial. *JAMA Cardiol* 2020;**5**:567–75. doi:10.1001/jamacardio.2019.6161

45 Reif J, Chan D, Jones D, *et al.* Effects of a Workplace Wellness Program on Employee Health, Health Beliefs, and Medical Use: A Randomized Clinical Trial. *JAMA Intern Med* 2020;**180**:952–60. doi:10.1001/jamainternmed.2020.1321

46 Patel A, Praveen D, Maharani A, *et al.* Association of Multifaceted Mobile Technology-Enabled Primary Care Intervention with Cardiovascular Disease Risk Management in Rural Indonesia. *JAMA Cardiol* 2019;**4**:978–86. doi:10.1001/jamacardio.2019.2974

47 Persell SD, Peprah YA, Lipiszko D, *et al.* Effect of Home Blood Pressure Monitoring via a Smartphone Hypertension Coaching Application or Tracking Application on Adults With Uncontrolled Hypertension: A Randomized Clinical Trial. *JAMA Netw open* 2020;**3**:e200255. doi:10.1001/jamanetworkopen.2020.0255

For peer review only - http://bmjopen.bmj.com/site/about/guidelines.xhtml

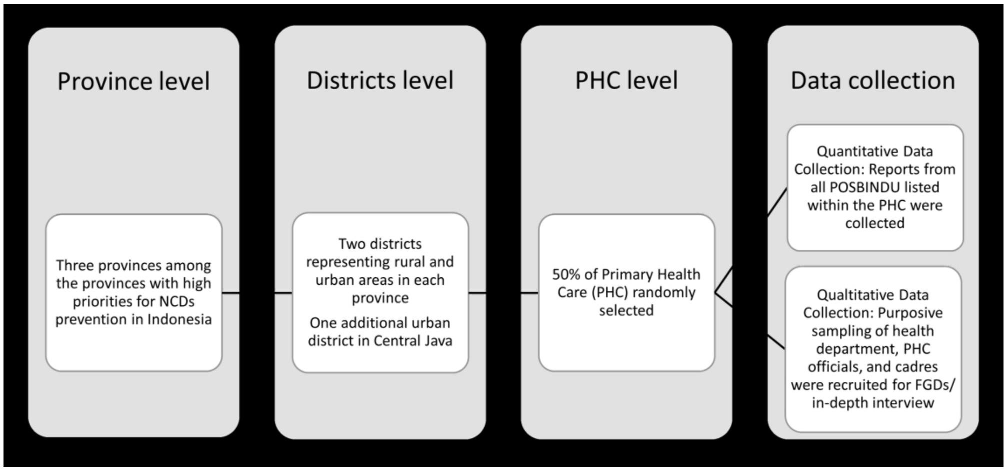

Figure 1. Study Sample Selection

258x119mm (300 x 300 DPI)

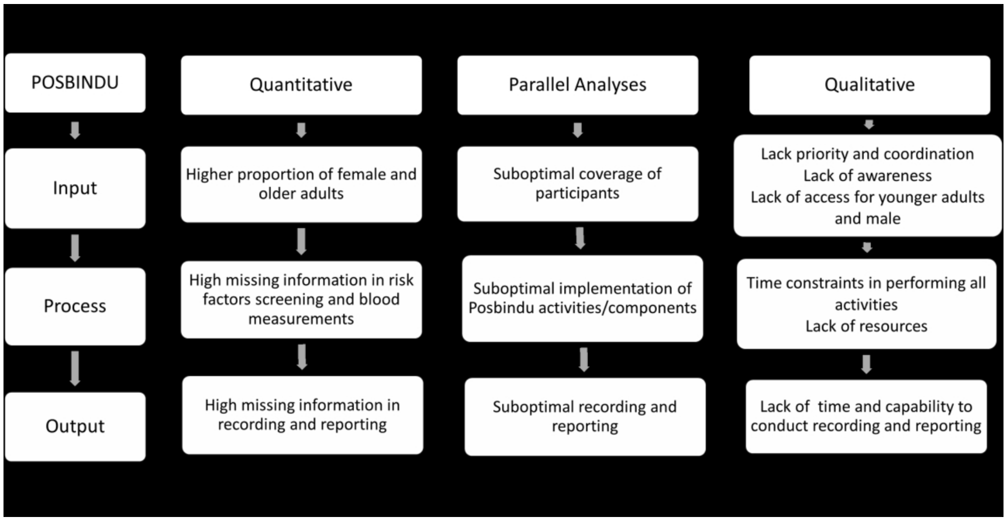

Figure 2. Synthesis of the Quantitative and Qualitative Findings

240x123mm (300 x 300 DPI)

STROBE Statement—Checklist of items that should be included in reports of ***cross-sectional studies***

	Item No	Recommendation	Page No
**Title and abstract**	1	(*a*) Indicate the study's design with a commonly used term in the title or the abstract	Page 1
		(*b*) Provide in the abstract an informative and balanced summary of what was done and what was found	Page 1
**Introduction**			
Background/rationale	2	Explain the scientific background and rationale for the investigation being reported	Page 2
Objectives	3	State specific objectives, including any prespecified hypotheses	Page 2-3
**Methods**			
Study design	4	Present key elements of study design early in the paper	Page 3
Setting	5	Describe the setting, locations, and relevant dates, including periods of recruitment, exposure, follow-up, and data collection	Page 3
Participants	6	(*a*) Give the eligibility criteria, and the sources and methods of selection of participants	Page 3
Variables	7	Clearly define all outcomes, exposures, predictors, potential confounders, and effect modifiers. Give diagnostic criteria, if applicable	Page 4
Data sources/ measurement	8*	For each variable of interest, give sources of data and details of methods of assessment (measurement). Describe comparability of assessment methods if there is more than one group	*Page 4*
Bias	9	Describe any efforts to address potential sources of bias	N/A, descriptive analyses
Study size	10	Explain how the study size was arrived at	Page 3
Quantitative variables	11	Explain how quantitative variables were handled in the analyses. If applicable, describe which groupings were chosen and why	Page 4
Statistical methods	12	(*a*) Describe all statistical methods, including those used to control for confounding	Page 4
		(*b*) Describe any methods used to examine subgroups and interactions	Page 4
		(*c*) Explain how missing data were addressed	Page 4
		(*d*) If applicable, describe analytical methods taking account of sampling strategy	N/A
		(*e*) Describe any sensitivity analyses	N/A
**Results**			
Participants	13*	(a) Report numbers of individuals at each stage of study—eg numbers potentially eligible, examined for eligibility, confirmed eligible, included in the study, completing follow-up, and analysed	Page 4
		(b) Give reasons for non-participation at each stage	N/A
		(c) Consider use of a flow diagram	Page 4 (referred)
Descriptive data	14*	(a) Give characteristics of study participants (eg demographic, clinical, social) and information on exposures and potential confounders	Page 5
		(b) Indicate number of participants with missing data for each variable of interest	Page 6

For peer review only - http://bmjopen.bmj.com/site/about/guidelines.xhtml

Outcome data	15*	Report numbers of outcome events or summary measures	Page 6
Main results	16	(*a*) Give unadjusted estimates and, if applicable, confounder-adjusted estimates and their precision (eg, 95% confidence interval). Make clear which confounders were adjusted for and why they were included	N/A, descriptive analyses
		(*b*) Report category boundaries when continuous variables were categorized	Page 5-6
		(*c*) If relevant, consider translating estimates of relative risk into absolute risk for a meaningful time period	N/A
Other analyses	17	Report other analyses done—eg analyses of subgroups and interactions, and sensitivity analyses	N/A
**Discussion**			
Key results	18	Summarise key results with reference to study objectives	Page 10
Limitations	19	Discuss limitations of the study, taking into account sources of potential bias or imprecision. Discuss both direction and magnitude of any potential bias	Page 11
Interpretation	20	Give a cautious overall interpretation of results considering objectives, limitations, multiplicity of analyses, results from similar studies, and other relevant evidence	Page 11
Generalisability	21	Discuss the generalisability (external validity) of the study results	Page 12
**Other information**			
Funding	22	Give the source of funding and the role of the funders for the present study and, if applicable, for the original study on which the present article is based	Page 12

*Give information separately for exposed and unexposed groups.

Note: An Explanation and Elaboration article discusses each checklist item and gives methodological background and published examples of transparent reporting. The STROBE checklist is best used in conjunction with this article (freely available on the Web sites of PLoS Medicine at http://www.plosmedicine.org/, Annals of Internal Medicine at http://www.annals.org/, and Epidemiology at http://www.epidem.com/). Information on the STROBE Initiative is available at www.strobe-statement.org.

For peer review only

For peer review only - http://bmjopen.bmj.com/site/about/guidelines.xhtml

Standards for Reporting Qualitative Research (SRQR)*

http://www.equator-network.org/reporting-guidelines/srqr/

Page/line no(s).

Title and abstract

**Title** - Concise description of the nature and topic of the study Identifying the study as qualitative or indicating the approach (e.g., ethnography, grounded theory) or data collection methods (e.g., interview, focus group) is recommended	Page 1/Line 1
**Abstract** - Summary of key elements of the study using the abstract format of the intended publication; typically includes background, purpose, methods, results, and conclusions	Page 1/Line 14-36

Introduction

**Problem formulation** - Description and significance of the problem/phenomenon studied; review of relevant theory and empirical work; problem statement	Page 2/Line 53-77
**Purpose or research question** - Purpose of the study and specific objectives or questions	Page 2/Line 80-82

Methods

**Qualitative approach and research paradigm** - Qualitative approach (e.g., ethnography, grounded theory, case study, phenomenology, narrative research) and guiding theory if appropriate; identifying the research paradigm (e.g., postpositivist, constructivist/ interpretivist) is also recommended; rationale**	Page 2/Line 98
**Researcher characteristics and reflexivity** - Researchers' characteristics that may influence the research, including personal attributes, qualifications/experience, relationship with participants, assumptions, and/or presuppositions; potential or actual interaction between researchers' characteristics and the research questions, approach, methods, results, and/or transferability	Page 3/ Line 116-119
**Context** - Setting/site and salient contextual factors; rationale**	Page 3/ Line 85-93
**Sampling strategy** - How and why research participants, documents, or events were selected; criteria for deciding when no further sampling was necessary (e.g., sampling saturation); rationale**	Page 3/ Line 114-121
**Ethical issues pertaining to human subjects** - Documentation of approval by an appropriate ethics review board and participant consent, or explanation for lack thereof; other confidentiality and data security issues	Page 3/Line 100-102
**Data collection methods** - Types of data collected; details of data collection procedures including (as appropriate) start and stop dates of data collection and analysis, iterative process, triangulation of sources/methods, and modification of procedures in response to evolving study findings; rationale**	Page 3/ Line 114-125

For peer review only - http://bmjopen.bmj.com/site/about/guidelines.xhtml

**Data collection instruments and technologies** - Description of instruments (e.g., interview guides, questionnaires) and devices (e.g., audio recorders) used for data collection; if/how the instrument(s) changed over the course of the study	Page 3/Line 148-150
**Units of study** - Number and relevant characteristics of participants, documents, or events included in the study; level of participation (could be reported in results)	Page 3/ Line 114-116
**Data processing** - Methods for processing data prior to and during analysis, including transcription, data entry, data management and security, verification of data integrity, data coding, and anonymization/de-identification of excerpts	Page 3/ Line 156-167
**Data analysis** - Process by which inferences, themes, etc., were identified and developed, including the researchers involved in data analysis; usually references a specific paradigm or approach; rationale**	Page 3/ Line 158-160
Techniques to enhance trustworthiness - Techniques to enhance trustworthiness and credibility of data analysis (e.g., member checking, audit trail, triangulation); rationale**	Page 3/ Line 160/163

Results/findings

**Synthesis and interpretation** - Main findings (e.g., interpretations, inferences, and themes); might include development of a theory or model, or integration with prior research or theory	Page 7/ Line 209-215
**Links to empirical data** - Evidence (e.g., quotes, field notes, text excerpts, photographs) to substantiate analytic findings	Page 8/Line 218-261

Discussion

**Integration with prior work, implications, transferability, and contribution(s) to the field** - Short summary of main findings; explanation of how findings and conclusions connect to, support, elaborate on, or challenge conclusions of earlier scholarship; discussion of scope of application/generalizability; identification of unique contribution(s) to scholarship in a discipline or field	Page 10/Line 279-319
**Limitations** - Trustworthiness and limitations of findings	Page 11/Line 353-356

Other

**Conflicts of interest** - Potential sources of influence or perceived influence on study conduct and conclusions; how these were managed	Page 12/Line 386
**Funding** - Sources of funding and other support; role of funders in data collection, interpretation, and reporting	Page 12/Line 381-383

*The authors created the SRQR by searching the literature to identify guidelines, reporting standards, and critical appraisal criteria for qualitative research; reviewing the reference lists of retrieved sources; and contacting experts to gain feedback. The SRQR aims to improve the transparency of all aspects of qualitative research by providing clear standards for reporting qualitative research.

2

For peer review only

**The rationale should briefly discuss the justification for choosing that theory, approach, method, or technique rather than other options available, the assumptions and limitations implicit in those choices, and how those choices influence study conclusions and transferability. As appropriate, the rationale for several items might be discussed together.

Reference:

O'Brien BC, Harris IB, Beckman TJ, Reed DA, Cook DA. **Standards for reporting qualitative research: a synthesis of recommendations.** *Academic Medicine*, Vol. 89, No. 9 / Sept 2014 DOI: 10.1097/ACM.0000000000000388

For peer review only

BMJ Open

Missed Opportunities in Hypertension Risk Factors Screening in Indonesia: A Mixed-methods Evaluation of Integrated Health Post (POSBINDU) Implementation

Journal:	*BMJ Open*
Manuscript ID	bmjopen-2021-051315.R2
Article Type:	Original research
Date Submitted by the Author:	27-Jan-2022
Complete List of Authors:	Widyaningsih, Vitri; Universitas Sebelas Maret, Public Health<br>Febrinasari, Ratih; Universitas Sebelas Maret, Pharmacology<br>Pamungkasari, Eti; Universitas Sebelas Maret, Public Health<br>Mashuri, Yusuf; Universitas Sebelas Maret, Parasitology<br>Sumardiyono, Sumardiyono; Universitas Sebelas Maret, Public Health<br>Balgis, Balgis; Universitas Sebelas Maret, Public Health<br>Koot, Jaap; University Medical Centre Groningen, Department of Health Sciences<br>Landsman-Dijkstra, Jeanet; University Medical Centre Groningen, Department of Health Sciences<br>Probandari, Ari; Universitas Sebelas Maret,
**Primary Subject Heading**:	Public health
Secondary Subject Heading:	Global health, Epidemiology, Health services research
Keywords:	Hypertension < CARDIOLOGY, International health services < HEALTH SERVICES ADMINISTRATION & MANAGEMENT, PUBLIC HEALTH, Quality in health care < HEALTH SERVICES ADMINISTRATION & MANAGEMENT

SCHOLARONE™
Manuscripts

I, the Submitting Author has the right to grant and does grant on behalf of all authors of the Work (as defined in the below author licence), an exclusive licence and/or a non-exclusive licence for contributions from authors who are: i) UK Crown employees; ii) where BMJ has agreed a CC-BY licence shall apply, and/or iii) in accordance with the terms applicable for US Federal Government officers or employees acting as part of their official duties; on a worldwide, perpetual, irrevocable, royalty-free basis to BMJ Publishing Group Ltd ("BMJ") its licensees and where the relevant Journal is co-owned by BMJ to the co-owners of the Journal, to publish the Work in this journal and any other BMJ products and to exploit all rights, as set out in our licence.

The Submitting Author accepts and understands that any supply made under these terms is made by BMJ to the Submitting Author unless you are acting as an employee on behalf of your employer or a postgraduate student of an affiliated institution which is paying any applicable article publishing charge ("APC") for Open Access articles. Where the Submitting Author wishes to make the Work available on an Open Access basis (and intends to pay the relevant APC), the terms of reuse of such Open Access shall be governed by a Creative Commons licence – details of these licences and which Creative Commons licence will apply to this Work are set out in our licence referred to above.

Other than as permitted in any relevant BMJ Author's Self Archiving Policies, I confirm this Work has not been accepted for publication elsewhere, is not being considered for publication elsewhere and does not duplicate material already published. I confirm all authors consent to publication of this Work and authorise the granting of this licence.

For peer review only

Missed Opportunities in Hypertension Risk Factors Screening in Indonesia: A Mixed-methods Evaluation of Integrated Health Post (POSBINDU) Implementation

Vitri Widyaningsih[1] , Ratih Puspita Febrinasari[1], Eti Poncorini Pamungkasari [1], Yusuf Ari Mashuri[1], Sumardiyono[1], Balgis[1], Jaap Koot[2], Jeanet Landsman-Dijkstra[2], Ari Probandari [1] on behalf of Scaling Up Non-Communicable Disease Intervention in South East Asia (SUNISEA) Project

[1]Faculty of Medicine, Universitas Sebelas Maret, Surakarta, Indonesia

[2] University of Groningen, University Medical Center Groningen, Department of Health Sciences, The Netherlands

Corresponding author: Prof. dr. Ari Natalia Probandari, MPH., Ph.D. Faculty of Medicine Universitas Sebelas Maret Surakarta, 57126, Central Java, Indonesia ari.probandari@staff.uns.ac.id
Phone: (0271) 646994

For peer review only

Abstract

Objectives: To assess the implementation and contextual barriers of POSBINDU, a community-based activity focusing on screening of Non-Communicable Diseases (NCDs), mainly hypertension and diabetes, in Indonesia.

Design: This was a concurrent mixed-methods study, with a cross-sectional analysis of secondary data and Focus Group Discussions on stakeholder of POSBINDU.

Setting: The study was conducted in seven districts in three provinces in Indonesia, with approximately 50% of the Primary Health Care (PHC) were selected as areas for data collection (n PHC=100).

Participants: From 475 POSBINDU sites, we collected secondary data from 54,224 participants. For the qualitative approach, 21 focus group discussions (FGDs) and 2 in-depth interviews were held among a total of 223 informants.

Primary outcomes and measures: Proportion of POSBINDU visitors getting the hypertension screening and risk factors' assessment, and barriers of POSBINDU implementation.

Results: Out of the 114,581 POSBINDU visits by 54,224 participants, most (80%) were female and adults over 50 years old (50%) showing a suboptimal coverage of male and younger adults. Approximately 95.1% of visitors got their blood pressure measured during their first visit; 35.3% of whom had elevated blood pressure. Less than 25% of the visitors reported to be interviewed for NCDs risk factors during their first visit, less than 80% had anthropometric measurements, and less than 15% had blood cholesterol examinations. We revealed lack of resources and limited time to perform the complexities of activities and reporting as main barrier for effective hypertension screening in Indonesia.

Conclusions: This study showed missed opportunities in hypertension risk factors screening in Indonesia. The barriers include a lack of access and implementation barriers (capability, resources, and protocols).

Keywords: Non-communicable diseases, hypertension, screening, community-based program

Article Summary

Findings

For peer review only - http://bmjopen.bmj.com/site/about/guidelines.xhtml

In a mixed-methods study, we found suboptimal implementation of POSBINDU which reflected the missed opportunities in screening for hypertension and its risk factors in Indonesia. Several barriers include suboptimal coverage, complexities of activities and overlap between different NCD-related programs, and lack of resources.

Implications

There is a need to improve coverage and implementation of POSBINDU for screening for hypertension and its risk factors. An integrated approach to improve the implementation of hypertension screening, from guidelines to practice is crucial.

Strengths and Limitations of this study

- This was a relatively large evaluation of POSBINDU in Indonesia, with almost two years of data.
- The findings from mixed-methods study provide more comprehensive information on POSBINDU implementation
- Information on the contextual factors of POSBINDU implementation can provide insights into steps to improve POSBINDU in the communities.
- The use of secondary data poses variations in blood pressure and anthropometrics measurements.
- The study limitation also includes the difficulty in differentiating whether the missed reporting was due to lack of activities or lack of reporting. Nevertheless, both the activities and reporting are important in NCDs screening, particularly in the follow up.

Introduction

The increasing trends of Non-Communicable Diseases (NCDs) in the world, including Indonesia, require targeted and specific primary and secondary prevention.[1,2] Hypertension, one of the most common NCDs, has a relatively high (33.4%) prevalence in Indonesia.[3][4] This figure is estimated to increase even further with the changing (more sedentary) lifestyle, unhealthy diet, rising prevalence of obesity, and the increasing life expectancy.[5] In 2015, hypertension attributed to 41% of all disability-adjusted life-years (DALYs) lost, and was the leading risk factor for cardiovascular diseases.[6] Economically, hypertension accounts for $370 billion in medical costs per year worldwide.[7] Major modifiable risk factors for NCDs include smoking, alcohol consumption, unhealthy diet and obesity, and a sedentary lifestyle.[8,9] With the heavy burden and the economic cost of this disease, primary and secondary prevention for hypertension and its risk factors become very important.

In 2010, the WHO has recommended the implementation of Package of Essential Interventions for Non-Communicable (PEN) Diseases for low- and middle-income countries.[10] In response, the Ministry of Health (MOH) in Indonesia launched the Integrated Health Post (POSBINDU), as part of the PEN program. POSBINDU, a community-based program for hypertension screening and prevention[11], was added to the several existing NCD-related programs Indonesia. These include Prolanis (Program Pengendalian Penyakit Kronis), a community-based hypertension and diabetes management program affiliated with primary care[12] and Posyandu Lansia, a community-based NCDs screening and management for the elderly.[13] Despite these efforts, the awareness and control of hypertension are still relatively low: only 25% of people with elevated blood pressure are aware of their condition, and only 54% of people diagnosed with hypertension take routine medication.[4,14,15] These conditions are still below the "rule of halves" for hypertension management, which recommends that 50% of hypertension patients be aware of their condition, with half of whom should be treated.[16,17]

For peer review only - http://bmjopen.bmj.com/site/about/guidelines.xhtml

A process evaluation is important in assessing the implementation, to identify barriers, and provide specific recommendations for improvement of POSBINDU. Previous studies have evaluated the effectiveness of the POSBINDU implementation.[11,18] However, they were lacking on the evaluation of contextual barriers in POSBINDU implementation. This study aims to portray the implementation of POSBINDU and its contextual barriers, to provide recommendations for better hypertension and its risk factors screening, and optimal linkage to care in Indonesia.

Methods

Setting

POSBINDU is a community-based activity run by community health cadres (volunteers) and supervised by Primary Health Care (PHC) officials. POSBINDU aims to empower communities in screening for NCDs and the risk factors, targeting individuals above 15 years old, particularly those of productive age.[19,20] The main activities include screening for NCDs (mainly hypertension and diabetes) and the risk factors (i.e., smoking, diet, physical activity, obesity). Further, POSBINDU also provides health education and facilitate referral to PHC.[19] For this study, we focus on POSBINDU implementation in screening of hypertension and its risk factor, particularly, since only 30% of hypertensive patients in Indonesia received formal diagnosis.[15]

Study Design

This was a concurrent mixed-methods study in seven districts in three provinces in Indonesia (Central Java, East Java, and North Sumatra). We purposely selected provinces with relatively high prevalence of NCDs based on a national health survey conducted in 2018.[21] Cross-sectional study by obtaining POSBINDU reports were conducted for the quantitative evaluation, whereas case study was conducted to explore barriers of POSBINDU implementation.

Ethnical approval

The study was approved by the ethical review board at Universitas Gadjah Mada, reference number KE/FK/0648/2019. The participants of Focus Group Discussion gave informed consent before participating in this study.

Data Collection

Within every one of the three provinces, we selected two districts: one city representing urban communities, and one district representing rural communities. In Central Java, an additional city was also selected (Figure 1). The rural/urban classification is based on population density and facilities available in the communities. For each district, approximately 50% of the Primary Health Care (PHC) were selected as areas for data collection (n PHC=100). Within the PHC, we collected data for quantitative process evaluation from all active POSBINDU in the areas (n POSBINDU=475). Due to the different number of POSBINDU within each district or PHCs, the number of POSBINDU visitors as well as visits varies by the areas. In most POSBINDU, online/electronic data were not available; Hence, data on participation were manually collected from the POSBINDU register. Data from 2018-2019 were collected, except for Central Java, in which data were available through September 2019.

For the qualitative approach, 21 focus group discussions (FGDs) and 2 in-depth interviews were held among a total of 223 informants: 22 from Districts Health Department, 101 from Primary Health Care (PHC) facilities and 100 POSBINDU cadres. The two in-depth interviews were conducted with health districts department officials. Within each district, we conducted purposive sampling to recruit health officials responsible for POSBINDU program from the district's health department, and primary health care. We also recruit 2-3 cadres from each PHC based on list of cadres obtained from PHC officials. These participants were recruited to obtain information on POSBINDU implementation facilitators and barriers. The size of the FGDs was on average 10 persons (min 4, max 18). Verbatim transcripts of the

For peer review only - http://bmjopen.bmj.com/site/about/guidelines.xhtml

FGD's were made for qualitative analyses. The FGD facilitators had public health background and experience in conducting qualitative research. All facilitators attended the preparatory meeting to discuss the FGDs and interview guidelines, to obtain similar perception regarding the aims of FGDs and interviews and items of the FGD guidelines.

Insert Figure 1 here

Outcome and variables measurements

Missed opportunities in hypertension screening were quantified by the proportion of POSBINDU visitors getting the risk factors anamnesis, and measurement of anthropometric tests, blood pressure and cholesterol. Analyses was conducted on each indicator to provide more detailed information on specific components of screening which were lacking. Sociodemographic variables which were available on the POSBINDU register, were included in the analyses: sex, age, and level of education. Age was classified into several groups based the Indonesian Ministry of Health classification for age (youth = 15-24 years old, adult = 25-44 years old, pre-elderly = 45-59 years old, and elderly => 60 years old). Occupation was not included in the analyses due to high missing value in the POSBINDU reports (>60%).

Personal and family history of NCDs were also obtained, which include seven (7) diseases: hypertension, diabetes, heart disease, stroke, asthma, cancer, and high blood cholesterol. Complete personal/family history variables were coded 1 if all information was available and coded 0 if at least one of the disease histories was missing. Any personal/family history variables were coded 1 if at least one of the disease histories was available and coded 0 if all of the history information was missing.

We also generate variable "incomplete information" which represent whether the individual received the recommended procedure (history taking, anthropometric measurement, blood pressure measurement, and blood examination). The proportion presented in the analyses, described the individuals who did not receive the complete recommended procedure.

We used the logic model framework for process evaluation to assess the implementation of POSBINDU. We adopted several indicators from the current literature on the use of logic model in process evaluation of community-based health intervention. [22–24] The FGDs theme as well as indicators of the secondary data developed based on the literature, were discussed with officials from health department and PHC officials in one pilot site for finalization. We further explored the barriers of POSBINDU implementation using a qualitative approach.

Analyses

Statistical analyses were conducted using STATA, to calculate the proportion of activities and outcomes. We further conduct Chi-square, T-test, and ANOVA to assess the statistical significance of the differences. Analyses were conducted on missing information, reflecting whether specific procedure in POSBINDU were carried out and reported. Further analyses on proportion of hypertension and BMI status were also conducted. The two indicators were reported due to relatively high availability of these data (92% and 76%) compared to other indicators. Verbatim transcript from FGDs and in-depth interviews recordings were analysed. Content analysis was applied for the qualitative data to ascertain barriers for the POSBINDU implementation in Indonesia by two independent researchers. To enhance trustworthiness, we assess barriers of POSBINDU from several sources for triangulation purposes: health and PHC officials to reflect implementer's perspective, and cadres to reflect implementers and users' perspective. During data analyses, we also discuss the findings with representative of the FGD participants, i.e., member checking. Parallel analyses were conducted to synthesize the findings from the quantitative and qualitative approaches. Weaving

For peer review only - http://bmjopen.bmj.com/site/about/guidelines.xhtml

technique, analysing the quantitative and qualitative findings together by theme or concept, was used to integrate the findings.[25]

Patients and public involvement

Patients or the public were not directly involved in the design, or conduct, or reporting, or dissemination plans of our research.

Results

Participation of community for hypertension screening in POSBINDU

Data from 114,581 POSBINDU visits (54,224 participants) were analysed. The findings showed similar patterns in the districts and provinces: more female and elderly participants. Approximately 80% were female participants, with the highest proportion of female participants in rural North Sumatra (95.5%). Meanwhile, in Java, a higher proportion of female participants were observed in urban areas (Table 1).

Table 1. Characteristics of POSBINDU Participants within the Three Provinces in Indonesia (POSBINDU Register, 2018-2019)

Characteristics	North Sumatra		East Java		Central Java		
	Rural	Urban	Rural	Urban	Rural	Urban	Total
Number of individuals	5,103	10,999	23,053	4,983	3,398	6,688	54,224
Number of PHC	11	23	29	9	11	17	100
Number of POSBINDU	38	38	283	27	27	62	475
Categorical (%, SE)							
Female	95.5 (0.3)	71.3 (0.4)	76.2 (0.3)	86.7 (0.5)	73.7 (0.8)	88.2 (0.4)	79.4 (0.2)
Age							
15-24	8.7 (0.4)	6.1 (0.3)	6.2 (0.2)	7.8 (0.4)	13.3 (0.7)	3.8 (0.3)	6.7 (0.1)
25-44	38.1 (0.8)	24.9 (0.5)	33.0 (0.3)	48.7 (0.8)	47.7 (1.0)	28.9 (0.7)	22.2 (0.2)
45-59	30.7 (0.7)	37.6 (0.6)	33.2 (0.3)	31.0 (0.6)	30.1 (0.9)	42.4 (0.7)	24.8 (0.2)
>60	22.4 (0.6)	31.3 (0.6)	27.6 (0.3)	12.5 (0.5)	8.9 (0.6)	24.9 (0.6)	24.7 (0.2)
Education**							
PS	2.6 (0.2)	15.3 (0.3)	57.3 (0.3)	50.5 (0.7)	41.1 (0.8)	25.2 (0.5)	38.0 (0.2)
HS	0.3 (0.07)	0.7 (0.1)	0.3 (0.0)	1.3 (0.2)	2.3 (0.3)	1.8 (0.2)	0.8 (0.0)
Univ	0.0 (0.0)	2.7 (0.2)	0.4 (0.04)	4.9 (0.3)	4.9 (0.4)	2.1 (0.2)	1.8 (0.1)
Missing	97.1 (0.2)	81.4 (0.4)	41.9 (0.3)	43.3 (0.7)	51.8 (0.9)	70.8 (0.6)	59.4 (0.2)
Number of visits							
1 time	87.0 (0.4)	77.4 (0.3)	68.4 (0.3)	65.6 (0.7)	84.5 (0.6)	56.4 (0.6)	71.2 (0.1)
2-6 times	12.9 (0.5)	21.7 (0.3)	21.1 (0.2)	23.9 (0.6)	13.3 (0.6)	35.1 (0.6)	22.0 (0.1)
7-12 times	0.1 (0.0)	0.6 (0.1)	6.3 (0.2)	6.0 (0.3)	2.1 (0.2)	5.9 (0.3)	5.3 (0.1)
>12 times	0 (0.0)	0.2 (0.1)	4.1 (0.1)	4.5 (0.3)	0.1 (0.0)	2.6 (0.2)	1.4 (0.1)
Continuous (mean, SE)							
Age	46.4 (0.2)	51.7 (0.2)	49.7 (0.1)	43.4 (0.2)	41.0 (0.3)	50.4 (0.2)	48.6 (0.8)
Number of visits	1.2 (0.8)	1.4 (0.1)	2.5 (0.2)	2.7 (0.5)	1.4 (0.2)	2.5 (0.3)	2.1 (0.1)

Notes:
Within province, rural-urban comparisons are significant at 0.05

For peer review only - http://bmjopen.bmj.com/site/about/guidelines.xhtml

For peer review only

Between provinces comparisons are significant at 0.05
Differences in proportion tested using Chi squares
SE= standard error
**Education = HS (High school) PS (Primary school/less) Univ (University/college), Missing = data missing

Despite the relatively high missing information on age (n missing= 12,084, or 22.3%), we found that the participants were on average of older age, with roughly 50% of participants aged over 45 years old, almost 25% were >60 years old (Table 1). The highest proportion of participants >60 years old were observed in rural East Java (31.3%), with mean age of 51.7 years old. We measured the youngest POSBINDU participants in rural Central Java (mean age 41.0 years old). Meanwhile, the missing information on education level were higher (almost 60%), with even higher proportion in North Sumatra.

In the span of the two years of secondary data collection, we found that, on average, the participants visit POSBINDU twice, with the lowest average of visits in North Sumatra (rounded to 1 visit/participant). Approximately 38,628 (71.2%) of participants visit POSBINDU once for two years, and 761 (1.4%) visits POSBINDU more than 12 times.

Table 2. Missing information and Risk Factors Characteristics within POSBINDU Participants (POSBINDU Register, 2018-2019)

Characteristics	North Sumatra		East Java		Central Java		
	Rural %(SE)	Urban %(SE)	Rural %(SE)	Urban %(SE)	Rural %(SE)	Urban %(SE)	Total %(SE)
Missing Information in All Visits							
n	6,061	15,774	57,504	13,422	4,925	16,895	114,581
Personal history (complete)	99.3 (0.1)	92.6 (0.2)	67.9 (0.2)	42.5 (0.4)	62.1 (0.7)	95.2 (0.2)	73.8 (0.1)
Family history (complete)	99.4 (0.1)	92.6 (0.2)	65.1 (0.2)	39.7 (0.4)	61.6 (0.7)	95.9 (0.2)	72.1 (0.1)
Personal history (any)	88.2 (0.4)	88.5 (0.3)	56.4 (0.2)	17.2 (0.3)	54.2 (0.7)	91.0 (0.2)	62.9 (0.1)
Family history (any)	97.5 (0.2)	88.9 (0.3)	57.3 (0.2)	28.9 (0.4)	53.5 (0.7)	93.8 (0.2)	65.7 (0.1)
Height	42.7 (0.6)	19.0 (0.2)	15.6 (0.2)	20.7 (0.3)	30.6 (0.7)	23.3 (0.3)	19.9 (0.1)
Weight measurement	35.8 (0.6)	16.0 (0.2)	18.8 (0.2)	5.6 (0.2)	8.9 (0.4)	12.3 (0.3)	16.4 (0.1)
Waist circumference	49.5 (0.6)	36.0 (0.3)	15.6 (0.2)	12.7 (0.2)	36.9 (0.6)	63.6 (0.4)	27.8 ().1)
Blood pressure	1.8 (0.2)	5.8 (0.2)	9.8 (0.1)	4.6 (0.2)	4.2 (0.3)	6.3 (0.2)	7.4 (0.1)
Blood cholesterol	87.0 (0.4)	80.1 (0.3)	81.9 (0.2)	97.4 (0.1)	91.1 (0.4)	82.0 (0.3)	84.2 (0.1)
Incomplete information	99.6 (0.1)	95.1 (0.2)	98.1 (0.1)	99.6 (0.1)	99.6 (0.1)	99.6 (0.1)	98.2 (0.1)
**Missing Information in First Visits**							
n	5,103	10,999	23,053	4,983	2,298	6,688	54,224
Personal history (complete)	99.2 (0.1)	89.9 (0.3)	72.8 (0.3)	35.1 (0.7)	49.3 (0.9)	92.6 (0.3)	76.3 (0.2)
Family history (complete)	99.3 (0.1)	89.8 (0.3)	71.3 (0.3)	41.4 (0.7)	50.9 (0.9)	93.5 (0.3)	76.4 (0.2)
Personal history (any)	88.2 (0.5)	85.1 (0.3)	58.3 (0.3)	15.3 (0.5)	40.1 (0.8)	86.9 (0.4)	68.3 (0.2)
Family history (any)	97.3 ().2)	85.2 (0.3)	60.6 (0.3)	30.0 (0.6)	40.1 (0.8)	90.5 (0.4)	65.0 (0.2)

For peer review only

For peer review only - http://bmjopen.bmj.com/site/about/guidelines.xhtml

Height	41.8 (0.7)	19.0 (0.4)	14.8 (0.2)	21.1 (0.6)	20.4 (0.7)	23.0 (0.5)	20.1 (0.2)
Weight	35.3 (0.3)	15.0 (0.3)	16.7 (0.2)	47.6 (0.3)	7.2 ().4)	12.9 (0.4)	15.9 (0.1)
Waist circumference	48.9 ().7)	33.6 (0.4)	11.1 (0.2)	12.9 (0.5)	23.0 ().7)	66.1 (0.6)	26.9 (0.2)
Blood pressure	1.7 (0.2)	5.4 (0.2)	4.9 (0.1)	5.0 (0.3)	3.2 (0.3)	7.0 (0.3)	4.9 (0.1)
Blood cholesterol	86.1 (0.5)	75.7 (0.4)	76.6 (0.3)	97.9 (0.2)	92.3 (0.4)	79.1 (0.5)	80.6 (0.2)
Incomplete information	99.6 (0.1)	93.0 (0.2)	96.7 (0.1)	99.7 (0.1)	99.4 (0.1)	99.4 (0.1)	97.01 (0.1)
**Risk Factors Screening in All Visits**							
**n**	3,423	12,015	45,108	10,484	3,374	12,750	87,154
BMI							
Normal	48.3 (0.9)	48.2 (0.5)	51.3 (0.2)	44.6 (0.5)	52.3 (0.9)	46.4 (0.4)	49.3 (0.2)
Underweight	4.9 (0.4)	4.5 (0.2)	8.0 (0.1)	4.5 (0.2)	9.1 (0.5)	3.8 (0.2)	6.3 (0.1)
Overweight	31.9 (0.8)	34.4 (0.4)	30.8 (0.2)	34.7 (0.4)	29.1 (0.8)	34.2 (0.4)	32.3 (0.2)
Obese	14.9 (0.6)	12.9 (0.3)	10.0 (0.3)	16.3 (0.4)	9.4 (0.5)	15.7 (0.3)	12.1 (0.1)
n	5,942	14,835	51,784	12,773	4,717	15,814	105,865
Hypertension	35.4 (0.6)	28.0 (0.4)	42.5 (0.2)	33.7 (0.4)	25.6 (0.6)	35.9 (0.4)	37.2 (0.1)
**Risk Factors Screening in First Visits**							
**n**	2,925	8,440	18,820	3,850	2,678	5,078	41,791
BMI							
Normal	49.3 (0.9)	48.0 (0.5)	51.5 (0.4)	45.3 (0.8)	52.4 (1.0)	44.4 (0.7)	49.3 (0.2)
Underweight	4.8 (0.4)	4.5 (0.2)	7.7 (0.2)	5.1 (0.4)	10.1 (0.6)	4.2 (0.3)	6.3 (0.1)
Overweight	32.0 (0.9)	34.2 (0.5)	30.4 (0.3)	33.3 (0.8)	28.7 (0.9)	35.1 (0.7)	32.0 (0.2)
Obese	13.9 (0.6)	13.3 (0.4)	10.4 (0.2)	16.3 (0.6)	8.8 (0.5)	16.3 (0.5)	12.4 (0.2)
n	5,008	10,379	21,858	4,725	3,288	6,201	51,459
Hypertension	34.5 (0.7)	28.5 (0.4)	40.5 (0.3)	31.6 (0.7)	25.1 (0.8)	37.9 (0.6)	35.3 (0.2)

Notes:
Within province, rural-urban comparisons are significant at 0.05
Between provinces comparisons are significant at 0.05
Differences in proportion tested using Chi squares
SE= standard error

We further observed the relatively high missing information for screening in POSBINDU across the districts, with the following general pattern. First, a relatively high proportion of missing information concerning the personal and family history, with East Java having the lowest proportion. Second, a relatively lower proportion of missing data on anthropometric measurements (less than 50%). Third, in all seven districts, the highest proportion of available data were for blood pressure measurements, followed by weight and height information. Last, our analysis identified higher missing values for blood cholesterol measurements (84,2%). For all measurements, there were significant differences between the three provinces, as well as between the rural and urban areas within the provinces (Table 2).

Based on available data, we found that obesity seems to be more prevalent in urban areas in Java, but relatively similar between rural and urban areas in North Sumatra. In contrast, hypertension was more prevalent in a rural area for East Java and North Sumatra but was more common in urban districts of Central Java (Table 2). However, these data should be interpreted cautiously due to the relatively high missing data on the measurements.

For peer review only - http://bmjopen.bmj.com/site/about/guidelines.xhtml

For peer review only

Barriers for the screening of hypertension in POSBINDU

The qualitative data supported the quantitative finding about lacking participation of male and younger population in POSBINDU. In the FGDs, cadres and health officials stated the barriers for male and younger participants to attend POSBINDU, including the inconvenience of POSBINDU schedule, as well as low awareness for hypertension screening (Table 3).

Table 3. Qualitative analyses of Focus Group Discussion amongst POSBINDU Cadres, Primary Health Care and Health Department Officials

Themes	Category	Codes
Suboptimal target population and gap in policy	Participants' characteristics	Younger adults rarely participate
		Lack of male participants
	Barrier to participations	Schedule incompatibility
		Low awareness for screening
		Lack of role model for screening
	Ineffective policy and coordination	Lack of prioritization for NCD
		Implementation gap of national policy/program at the local level
		The need for coordination with different stakeholders
		The need for coordination among NCD-related programs
Lack of human resources in terms of capability and quantity for hypertension screening	Cadres have multiple tasks, with time constraints	POSBINDU cadres often have to multitask and handling other community programs
		Cadres are volunteers with other obligations
	Cadres' competencies	Lack of knowledge on hypertension and other NCD
		Lack of ability to conduct measurements and provide health education
		Lack of ability to conduct recording and reporting
	Lack of NCD program officers for supervision and reporting	Lack of NCD program officers at PHC
		Most program officers are responsible for multiple tasks/programs
		Lack of reporting officers
	Provision of Referral Counselling	The participant with hypertension is not always referred to PHC
		Lack of counseling to participants before the referral made
		POSBINDU has referral form, but rarely used
		Treatment for the referral is covered by their health insurance
Lack of resources for hypertension screening and prevention	Equipment for hypertension screening	The equipment is sometimes incomplete
		Equipment maintenance is inadequate
		Limited logistics for cholesterol measurement
	Lack of budget	POSBINDU is funded by the government, stakeholder (private sectors) or community
		Lack of budget for POSBINDU activities

For peer review only - http://bmjopen.bmj.com/site/about/guidelines.xhtml

		Lack of budget for cadres training and incentives
	Health education material	Lack of health education materials
	Infrastructure for recording and reporting	Not all cadres have laptops
		Limited internet connection in some areas
		Most POSBINDU stations use manual reporting
Time constraints for implementation based on MOH standard	The complexity of activities and time limitation	The time required for examination is too long
		Too many information needs to be asked and filled out
		The referral form is rarely used
	The complexity of reporting forms	Many forms need to be filled, while time is limited
		A simplified form in checklist format is preferred

The need for role model from community leader to improve participation and the barrier for participation, particularly among males is highlighted by these quotes:

> *"Yes, we don't have a lot of men (participants), because they are working " (Cadre, FGD#21)*

> *"In our* POSBINDU*, the awareness for early screening is still low. Only several people come (to* POSBINDU*), younger people don't want to come because* (POSBINDU *is conducted) during working days"(Cadre, FGD#3)*

> *"...Socialization for this (POSBINDU) is needed, often, the community leader in our area don't want to participate because they are afraid to be screened" (Cadre, FGD#2)*

> *"... when I asked the communities, why they did not come to POSBINDU, or why there were only few people, they said because I (the community member) were not sick, so why do I need to get (health) check-up (?). So, they were not aware that POSBINDU is not only for those who are sick" (Health official, FGD#19)*

> "I *asked POSBINDU (participant), why elderly? Where are the younger population? And they said that the young stayed at home because they were embarrassed if they have diseases... "(Health official, FGD#16)*

Lack of priority and overlap of NCD-related programs also contribute to a suboptimal target population of POSBINDU, as illustrated by the following quote:

> "*(NCD) is not a priority program, hence, there's a lack of commitment between the superior (health department) with the program officials, for example." (Health official, FGD#7)*

> *"... (different department in) the Ministry of Health focus on specific diseases, such as diabetes and cancer... However, in the community, (the programs) become general. (We) run Polindes, (Posyandu) Lansia, POSBINDU, School Health Program (Types of community based public health programs in Indonesia). In my opinion, the regulation is rigid and detailed, but the implementation is mixed (overlap). If we want to give optimum results, it takes efforts." (Health official, FGD#10).*

Several barriers for implementation were revealed. The cadres and health officers often have to run several different programs. The FGDs also revealed a lack of capability of cadres to conduct measurements for hypertension screening, providing health education, and also conducting the

For peer review only - http://bmjopen.bmj.com/site/about/guidelines.xhtml

recording and reporting of the POSBINDU activities and measurements. The informants also mentioned a lack of resources, including budget, equipment, and logistics to conduct all the measurements.

> *"One person can hold 5 positions in PHC activities… POSBINDU cadres, Posyandu Lansia cadres, and other programs. "(Cadre, FGD#11)*

> *"(cadres of) POSBINDU do not have laptop nor cell phone for the reporting application (of* POSBINDU*), hence, we report to PHC manually" (Cadre, FGD#14)*

The barriers also include the complexities of the activities and measurements, as well as extensive reporting forms, which require a long time to be completed.

> *"…it takes a long time, because of the measurements and stages (of* POSBINDU *activities) " (PHC officer, FGD#8)*

> *"…*POSBINDU *report is too time-consuming, because it is long (detail), including identity, cell phone number, address, and others… and it has to be filled out every month." (PHC officer, FGD#6)*

Interestingly, in several districts, we found the implementation of mobile POSBINDU, moving from one community to the other within the same subdistricts.

> *"Our* POSBINDU *is mobile, we have ten communities, so every week, we move from one community to the next, focusing on people 15-59 years old." (Cadre, FGD#18)*

We further synthesized the quantitative and qualitative results. We categorised the barriers into three main parts: 1) input, reflecting the target population/coverage of POSBINDU, 2) process, describing the implementation of POSBINDU activities, and 3) output, reflecting the recording and reporting process of POSBINDU (Figure 2). Results show that in both approaches we found lacking participation of male and younger people in POSBINDU. Lack of priority for NCD screening and ineffective coordination among stakeholders, combined with lack of awareness and access might attribute to this finding. The high missed opportunity, particularly in history taking and measurements, were likely due to the complexity of the activities/measurements, as well as lack of resources. The high missing data also stem from the complexity of the forms and lack of capability for online reporting.

Insert Figure 2 here

Discussion

In this study, we revealed missed opportunities in input, activities, and output of POSBINDU implementation in screening for hypertension and its risk factors. Several contextual barriers were identified. The suboptimal coverage was possibly due to lack of priority for NCD screening, lack of awareness and access, and overlap of NCD-related program. The suboptimal activities and reporting were likely caused by a lack of resources, as well as limited time to perform the complexities of activities and reporting according to MOH guideline.

The missed opportunity to screen male and younger population that we found in this study is particularly concerning. Although the prevalence is lower than of the older population, hypertension prevalence among young Indonesian is still relatively high (28%).[26] While the target population of POSBINDU is listed as those 15 years or older, the elderly are usually targeted in Posyandu Lansia, a community-based screening and management for the elderly population.[27] Awareness is also lower in male and younger adults, signaling the need to screen this population.[3] Ideally, POSBINDU

For peer review only - http://bmjopen.bmj.com/site/about/guidelines.xhtml

becomes the "gatekeeper" for screening in the community. Hypertensive and diabetic patients were then referred to PHC and joined Prolanis, a community-based activity funded by the health insurance program, for management of chronic diseases patients.[12]

Furthermore, with a lack of male participation, POSBINDU is missing one of the key target populations for risk factors screening: smokers. Analysis of a national survey in 2014 reported 32% prevalence of smoking, with approximately 40% of males aged 15-55 years old and 14% of male adolescents are current smokers.[28–30] Further, 20% of Indonesia's total chronic diseases are attributed to smoking, with hypertension as the highest proportion.[31] Screening for hypertension and its risk factors earlier, combined with lifestyle-based interventions effectively avoid future complications.[32,33]

We also revealed the need to prioritise and reorganise the current NCD-related programs, to address the suboptimal coverage and the overlap. An example of the gap between the national recommendation and local implementation is reflected in the coordination of existing NCD-related programs: POSBINDU, Posyandu Lansia, and PANDU PTM. In the MOH, the PANDU PTM (Pelayanan Terpadu Penyakit Tidak Menular, Integrated Health Services for NCDs) and POSBINDU are regulated under the Directorate for Disease Management, while Posyandu Lansia is under the Directorate of Public Health. Despite the different directorates, the implementation at community level is often conducted simultaneously and often overlap. Reporting, however, is conducted separately. Hence, as previous studies have noted, we also recommend the need of comprehensive and coordinated NCDs prevention program in Indonesia.[34–36]

The relatively high missed opportunity in screening for hypertension risk factors, as well as sociodemographic characteristics found in this study, portrays suboptimal implementation of POSBINDU. This can be caused by a lack of recording and reporting (monitoring and evaluation fidelity) or lack of measurement (implementation fidelity). In our further elaboration during the FGDs, we found that lack of human resources might contribute to the suboptimal implementation of POSBINDU. Our findings revealed the need to train cadres to improve their skills and efficiency in conducting the measurements and history taking, as well as reporting the measurements. This is in line with findings from Meinema et al (2017 and Abdell-All et al (2018)[37,38]. Our findings also imply the complexities of the activities and reporting of POSBINDU which lead to ineffective implementation. It is important to ensure that valuable screening information can be recorded and followed up, for better intervention. A simplified screening program with integrated reporting is needed.

In this study, we also discovered lack of financial resources and equipment as barriers to POSBINDU implementation. The integration of POSBINDU and PANDU PTM to the national health insurance scheme might be important to ensure the sustainability of funding for the program. Integration of POSBINDU into the national health insurance can also improve participation of the working population, most of whom are covered by the national health insurance.[39] Previous studies have reported an increase in uptake of service by health insurance membership.[40–42]

Based on our findings, we identified two main areas that needs to be improved: coverage and implementation of POSBINDU. To improve coverage of POSBINDU, there are two important steps that we recommend. First, an integrated approach with collaboration amongst different programs and directorates to reduce the overlap and simplify the POSBINDU implementation at the PHC and community level. PANDU PTM as the adaptation of WHO PEN,[43] needs to be implemented in a wider scale. Second, redirecting the target population of hypertension screening, to cover also younger and male population. A workplace-based screening program which can address the barriers identified in the qualitative findings is recommended.[44,45] For this younger population, the use of mobile technology for monitoring of risk factors and measurement might be effective. Previous

For peer review only - http://bmjopen.bmj.com/site/about/guidelines.xhtml

studies have reported the effectiveness of mobile health for hypertension screening and risk stratification.[46,47]

To improve the implementation and components of POSBINDU activities, a simplified algorithm to screen and refer the target population is needed. The algorithm needs to be developed both in the electronic format and manual format to address the different capabilities of community cadres and resources in the community. Simplifying the program and reporting systems will also reduce the workload of PHC and district health officials. Further, a clear algorithm for the referral of "screened" cases to PHC is important. The readiness of the PHCs also needs to be improved to adequately manage the potential surge in referred cases. Lastly, there is a need to integrate hypertension and CVD screening program into the national health insurance system. Hence, ensuring the sustainability of funding and resources of the program. With these approaches, comprehensive screening for hypertension and CVD along the continuum of care might be more effective.

This study has several limitations. First, the proportion of our measures are not reflective for the whole target population of POSBINDU, since the participants were mostly female and of older age. The characteristics of our sample, which are generally older with a higher proportion of females, drive the proportion of risk factors higher than that of the general population in Indonesia. However, this study reflects the current participants of POSBINDU. Second, we used a secondary data collection by POSBINDU cadres, the high number of missing data that we presented in this study, probably stem from two main sources: omissions in reporting or a true lack in measurement/activities. Nevertheless, both the activities and reporting are important in NCDs screening, particularly in the follow-up. The secondary data also prone to measurement bias, particularly, with the variations in POSBINDU measurements by cadres. The Ministry of Health provided guidelines in the measurement for hypertension in POSBINDU, however, the implementation might vary. The high missing information on several sociodemographic characteristics i.e., occupation and education, also limit our ability to conduct multivariable analyses. Another limitation of this study is we have not included the perspective of POSBINDU participants in the FGDs. Instead, we considered the POSBINDU cadres to represents the voice of both the implementers as well as users. However, we include the perspective of the POSBINDU participants as users in the baseline of our prospective data collection (ongoing). The users' perspective can provide further insights into barriers and facilitators of POSBINDU implementation.

Despite the limitation, there are several strengths of the study: First, to our knowledge, this was the first relatively large evaluation of POSBINDU. Second, the use of a mixed-methods study design, and therefore, providing more comprehensive information on POSBINDU implementation. Third, the study also investigates the contextual factors that should be addressed in the improvement of the community-based hypertension screening program in Indonesia. This study might provide insights into POSBINDU implementation in other areas in Indonesia and can be the basis for further recommendation to improve POSBINDU implementation.

Conclusion

This study showed the suboptimal implementation of POSBINDU activities. Particularly, the missed opportunity in screening for hypertension risk factors in Indonesia. The barriers include a lack priority for NCDs, lack of awareness and access for subpopulation, and several implementation barriers: capability, resources, and protocols. An innovative approach to simplify and improve the capacity of POSBINDU is in preparation to optimize the screening and linkage to hypertension care in Indonesia. This study provides evidence-based recommendations in improving the current implementation of POSBINDU, in the Indonesian context.

For peer review only - http://bmjopen.bmj.com/site/about/guidelines.xhtml

Data Statement

The de-identified data from this study is available upon request to the corresponding or first author pending thorough review of request and adherence to the Indonesian government regulation on data sharing.

Author Contributions

VW, AP, RFP, EP, YM, JK and JLD contributed to the design of the study. VW, A, RFP, EP, YM, B, and S participate actively in study implementation, including in data collection. VW, YM, S, and RFP analyzed the quantitative data. AP, EP, and B analyzed the qualitative data. VW, AP, RFP, S, and YM draft the manuscript with all co-authors revised critically. All authors read and approved the final manuscript.

Funding

This work was supported by the Horizon 2020 research program of the European Union. This study was co-financed by the Horizon 2020 research program of the European Union, under grant 825026 Scaling Up NCDs interventions in Southeast Asia.

Conflict of Interest

None declared

License

The Corresponding Author has the right to grant on behalf of all authors and does grant on behalf of all authors, an exclusive licence (or non exclusive for government employees) on a worldwide basis to the BMJ Publishing Group Ltd ("BMJ"), and its Licencees to permit this article (if accepted) to be published in The BMJ's editions and any other BMJ products and to exploit all subsidiary rights, as set out in our licence.

Acknowledgment

We thank SUNISEA project consortium and the district health departments, primary health care staffs and cadres of POSBINDU in the three provinces, who have contributed to this study.

Figure 1. Study Sample Selection

Figure 2. Synthesis of the Quantitative and Qualitative Findings

References

1 Basu S, Millett C. Social epidemiology of hypertension in middle-income countries: Determinants of prevalence, diagnosis, treatment, and control in the WHO SAGE study. *Hypertension* 2013;**62**:18–26. doi:10.1161/HYPERTENSIONAHA.113.01374

2 Mills KT, Bundy JD, Kelly TN, *et al.* Global disparities of hypertension prevalence and control. *Circulation* 2016;**134**:441–50. doi:10.1161/CIRCULATIONAHA.115.018912

3 Hussain MA, Al Mamun A, Reid C, *et al.* Prevalence, awareness, treatment and control of hypertension in Indonesian adults aged ≥40 years: Findings from the Indonesia Family Life Survey (IFLS). *PLoS One* 2016;**11**:1–16. doi:10.1371/journal.pone.0160922

4 Peltzer K, Pengpid S. The Prevalence and Social Determinants of Hypertension among Adults in Indonesia: A Cross-Sectional Population-Based National Survey. *Int J Hypertens* 2018;**2018**. doi:10.1155/2018/5610725

5 Purnamasari D. The Emergence of Non-communicable Disease in Indonesia. *Acta Med Indones* 2018;**50**:273–4.http://www.ncbi.nlm.nih.gov/pubmed/30630990

6 Vos T, Abajobir AA, Abbafati C, *et al.* Global, regional, and national incidence, prevalence, and

For peer review only - http://bmjopen.bmj.com/site/about/guidelines.xhtml

years lived with disability for 328 diseases and injuries for 195 countries, 1990-2016: A systematic analysis for the Global Burden of Disease Study 2016. *Lancet* 2017;**390**:1211–59. doi:10.1016/S0140-6736(17)32154-2

7 Gaziano T. Prevention and treatment of chronic diseases in developing countries. 2011;:1–20.http://www.un.org/esa/population/publications/expertpapers/2011-2-gaziano.pdf

8 Allen L, Williams J, Townsend N, *et al.* Socioeconomic status and non-communicable disease behavioural risk factors in low-income and lower-middle-income countries: a systematic review. *Lancet Glob Heal* 2017;**5**:e277–89. doi:10.1016/S2214-109X(17)30058-X

9 Rosengren A, Smyth A, Rangarajan S, *et al.* Socioeconomic status and risk of cardiovascular disease in 20 low-income, middle-income, and high-income countries: the Prospective Urban Rural Epidemiologic (PURE) study. *Lancet Glob Heal* 2019;**7**:e748–60. doi:10.1016/S2214-109X(19)30045-2

10 WHO. Package of Essential Noncommunicable (PEN) Disease Interventions for Primary Health Care. *Geneva World Heal Organ* 2010;:1–66.papers2://publication/uuid/FC98856B-4122-4325-826F-20FA6B89DEBD

11 Ferdianto A, Tamtomo DG, Sulaeman ES. Does the Integrated Health Post have Contextual Effect on Tertiary Preventive Behavior among Hypertensive Patients? A Multilevel Analysis Evidence from Surakarta. *J Heal Promot Behav* 2019;**4**:224–34. doi:10.26911/thejhpb.2019.04.03.07

12 Khoe LC, Wangge G, Soewondo P, *et al.* The implementation of community-based diabetes and hypertension management care program in Indonesia. *PLoS One* 2020;**15**:1–9. doi:10.1371/journal.pone.0227806

13 Yamada M, Hapsari ED, Matsuo H. Behaviors toward noncommunicable diseases prevention and their relationship with physical health status among community-dwelling, middleaged and older women in Indonesia. *Int J Environ Res Public Health* 2020;**17**. doi:10.3390/ijerph17072332

14 Maharani A, Sujarwoto, Praveen D, *et al.* Cardiovascular disease risk factor prevalence and estimated 10-year cardiovascular risk scores in Indonesia: The SMARThealth Extend study. *PLoS One* 2019;**14**:1–13. doi:10.1371/journal.pone.0215219

15 Turana Y, Tengkawan J, Soenarta AA. Asian management of hypertension: Current status, home blood pressure, and specific concerns in Indonesia. *J Clin Hypertens* 2020;**22**:483–5. doi:10.1111/jch.13681

16 Scheltens T, Bots ML, Numans ME, *et al.* Awareness, treatment and control of hypertension: The 'rule of halves' in an era of risk-based treatment of hypertension. *J Hum Hypertens* 2007;**21**:99–106. doi:10.1038/sj.jhh.1002123

17 Chen R, Tunstall-Pedoe H, Morrison C, *et al.* Trends and social factors in blood pressure control in Scottish MONICA surveys 1986-1995: The rule of halves revisited. *J Hum Hypertens* 2003;**17**:751–9. doi:10.1038/sj.jhh.1001612

18 Alfiyah A, Pujiyanto P. an Analysis on the Implementation of the Integrated Guidance Post (Posbindu) Activities for Non-Communicable Diseases At Bogor City in 2018. *J Indones Heal Policy Adm* 2019;**4**:11–5. doi:10.7454/ihpa.v4i1.2388

19 Ministry of Health (MOH) - Kementerian Kesehatan. Petunjuk Teknis Pos Pembinaan Terpadu Penyakit Tidak Menular (Posbindu PTM). 2012. http://p2ptm.kemkes.go.id/uploads/2016/10/Petunjuk-Teknis-Pos-Pembinaan-Terpadu-

For peer review only - http://bmjopen.bmj.com/site/about/guidelines.xhtml

Penyakit-Tidak-Menular-POSBINDU-PTM-2013.pdf

20 Moeloek NF. Indonesia national health policy in the transition of disease burden and health insurance coverage. *Med J Indones* 2017;**26**:3–6. doi:10.13181/mji.v26i1.1975

21 Ministry of Health (MOH) - Kementerian Kesehatan. Riskesdas Main Results 2018. 2018. https://www.litbang.kemkes.go.id/hasil-utama-riskesdas-2018/

22 Smith JD, Li DH, Rafferty MR. The implementation research logic model: A method for planning, executing, reporting, and synthesizing implementation projects. *medRxiv* 2020;:1–12. doi:10.1101/2020.04.05.20054379

23 Sharma S, Adetoro OO, Vidler M, *et al.* A process evaluation plan for assessing a complex community-based maternal health intervention in Ogun State, Nigeria. *BMC Health Serv Res* 2017;**17**:1–10. doi:10.1186/s12913-017-2124-4

24 Wong ST, Yin D, Bhattacharyya O, *et al.* Developing a performance measurement framework and indicators for community health service facilities in urban China. *BMC Fam Pract* 2010;**11**. doi:10.1186/1471-2296-11-91

25 Fetters MD, Curry LA, Creswell JW. Achieving integration in mixed methods designs - Principles and practices. *Health Serv Res* 2013;**48**:2134–56. doi:10.1111/1475-6773.12117

26 Sudharsanan N, Geldsetzer P. Impact of coming demographic changes on the number of adults in need of care for hypertension in Brazil, China, India, Indonesia, Mexico, and South Africa: A modeling study. *Hypertension* 2019;**73**:770–6. doi:10.1161/HYPERTENSIONAHA.118.12337

27 Rahmawati R, Bajorek B V. Access to medicines for hypertension: A survey in rural Yogyakarta province, Indonesia. *Rural Remote Health* 2018;**18**. doi:10.22605/RRH4393

28 Amalia B, Cadogan SL, Prabandari YS, *et al.* Socio-demographic inequalities in cigarette smoking in Indonesia, 2007 to 2014. *Prev Med (Baltim)* 2019;**123**:27–33. doi:10.1016/j.ypmed.2019.02.025

29 Sujarwoto S. Sleep Disturbance in Indonesia: How Much Does Smoking Contribute? *Behav Sleep Med* 2019;**00**:1–14. doi:10.1080/15402002.2019.1682584

30 Kusumawardani N, Tarigan I, Suparmi, *et al.* Socio-economic, demographic and geographic correlates of cigarette smoking among Indonesian adolescents: results from the 2013 Indonesian Basic Health Research (RISKESDAS) survey. *Glob Health Action* 2018;**11**. doi:10.1080/16549716.2018.1467605

31 Kristina SA, Endarti D, Wiedyaningsih C, *et al.* Health Care Cost of Noncommunicable Diseases Related to Smoking in Indonesia, 2015. *Asia-Pacific J Public Heal* 2018;**30**:29–35. doi:10.1177/1010539517751311

32 Saif-Ur-Rahman KM, Hasan M, Hossain S, *et al.* Non-pharmacological interventions for the prevention of hypertension in low-income and middle-income countries: Protocol for a systematic review and meta-analysis. *BMJ Open* 2018;**8**:6–11. doi:10.1136/bmjopen-2017-020724

33 Pantell MS, Prather AA, Downing JM, *et al.* Association of Social and Behavioral Risk Factors With Earlier Onset of Adult Hypertension and Diabetes. *JAMA Netw open* 2019;**2**:e193933. doi:10.1001/jamanetworkopen.2019.3933

34 Kusuma D, Kusumawardani N, Ahsan A, *et al.* On the verge of a chronic disease epidemic: Comprehensive policies and actions are needed in Indonesia. *Int Health* 2019;**11**:422–4.

For peer review only

For peer review only - http://bmjopen.bmj.com/site/about/guidelines.xhtml

doi:10.1093/inthealth/ihz025

35 Mboi N, Murty Surbakti I, Trihandini I, *et al.* On the road to universal health care in Indonesia, 1990–2016: a systematic analysis for the Global Burden of Disease Study 2016. *Lancet* 2018;**392**:581–91. doi:10.1016/S0140-6736(18)30595-6

36 Jayanna K, Swaroop N, Kar A, *et al.* Designing a comprehensive Non-Communicable Diseases (NCD) programme for hypertension and diabetes at primary health care level: Evidence and experience from urban Karnataka, South India. *BMC Public Health* 2019;**19**:1–12. doi:10.1186/s12889-019-6735-z

37 Meinema JG, Haafkens JA, Jaarsma DADC, *et al.* Development and evaluation of a culturally appropriate hypertension education (CAHE) training program for health care providers. *PLoS One* 2017;**12**:1–13. doi:10.1371/journal.pone.0178468

38 Abdel-All M, Thrift AG, Riddell M, *et al.* Evaluation of a training program of hypertension for accredited social health activists (ASHA) in rural India. *BMC Health Serv Res* 2018;**18**:1–11. doi:10.1186/s12913-018-3140-8

39 Ng JYS, Ramadani RV, Hendrawan D, *et al.* National Health Insurance Databases in Indonesia, Vietnam and the Philippines. *PharmacoEconomics - Open* 2019;**3**:517–26. doi:10.1007/s41669-019-0127-2

40 Erlangga D, Ali S, Bloor K. The impact of public health insurance on healthcare utilisation in Indonesia: evidence from panel data. *Int J Public Health* 2019;**64**:603–13. doi:10.1007/s00038-019-01215-2

41 Sharma M, Teerawattananon Y, Luz A, *et al.* Institutionalizing Evidence-Informed Priority Setting for Universal Health Coverage: Lessons From Indonesia. *Inq (United States)* 2020;**57**. doi:10.1177/0046958020924920

42 Vidyattama Y, Miranti R, Resosudarmo BP. The Role of Health Insurance Membership in Health Service Utilisation in Indonesia. *Bull Indones Econ Stud* 2014;**50**:393–413. doi:10.1080/00074918.2014.980380

43 Ministry of Health (MOH) - Kementerian Kesehatan. Pandu PTM A Community on intervention model and control's of NCD's. 2017.

44 Wang Z, Wang X, Shen Y, *et al.* Effect of a Workplace-Based Multicomponent Intervention on Hypertension Control: A Randomized Clinical Trial. *JAMA Cardiol* 2020;**5**:567–75. doi:10.1001/jamacardio.2019.6161

45 Reif J, Chan D, Jones D, *et al.* Effects of a Workplace Wellness Program on Employee Health, Health Beliefs, and Medical Use: A Randomized Clinical Trial. *JAMA Intern Med* 2020;**180**:952–60. doi:10.1001/jamainternmed.2020.1321

46 Patel A, Praveen D, Maharani A, *et al.* Association of Multifaceted Mobile Technology-Enabled Primary Care Intervention with Cardiovascular Disease Risk Management in Rural Indonesia. *JAMA Cardiol* 2019;**4**:978–86. doi:10.1001/jamacardio.2019.2974

47 Persell SD, Peprah YA, Lipiszko D, *et al.* Effect of Home Blood Pressure Monitoring via a Smartphone Hypertension Coaching Application or Tracking Application on Adults With Uncontrolled Hypertension: A Randomized Clinical Trial. *JAMA Netw open* 2020;**3**:e200255. doi:10.1001/jamanetworkopen.2020.0255

For peer review only - http://bmjopen.bmj.com/site/about/guidelines.xhtml

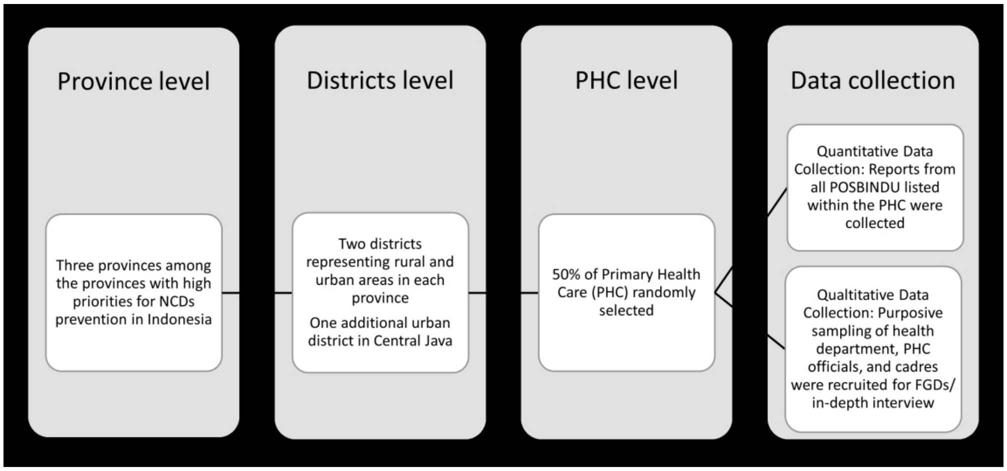

Figure 1. Study Sample Selection

258x119mm (330 x 330 DPI)

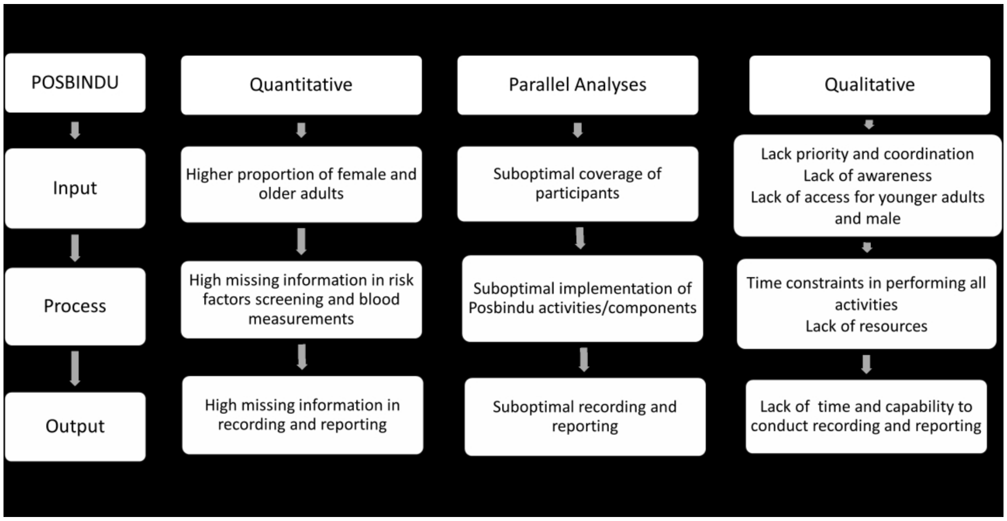

Figure 2. Synthesis of the Quantitative and Qualitative Findings

240x123mm (330 x 330 DPI)

STROBE Statement—Checklist of items that should be included in reports of ***cross-sectional studies***

	Item No	Recommendation	Page No
**Title and abstract**	1	(*a*) Indicate the study's design with a commonly used term in the title or the abstract	Page 1
		(*b*) Provide in the abstract an informative and balanced summary of what was done and what was found	Page 1
**Introduction**			
Background/rationale	2	Explain the scientific background and rationale for the investigation being reported	Page 2
Objectives	3	State specific objectives, including any prespecified hypotheses	Page 2-3
**Methods**			
Study design	4	Present key elements of study design early in the paper	Page 3
Setting	5	Describe the setting, locations, and relevant dates, including periods of recruitment, exposure, follow-up, and data collection	Page 3
Participants	6	(*a*) Give the eligibility criteria, and the sources and methods of selection of participants	Page 3
Variables	7	Clearly define all outcomes, exposures, predictors, potential confounders, and effect modifiers. Give diagnostic criteria, if applicable	Page 4
Data sources/ measurement	8*	For each variable of interest, give sources of data and details of methods of assessment (measurement). Describe comparability of assessment methods if there is more than one group	*Page 4*
Bias	9	Describe any efforts to address potential sources of bias	N/A, descriptive analyses
Study size	10	Explain how the study size was arrived at	Page 3
Quantitative variables	11	Explain how quantitative variables were handled in the analyses. If applicable, describe which groupings were chosen and why	Page 4
Statistical methods	12	(*a*) Describe all statistical methods, including those used to control for confounding	Page 4
		(*b*) Describe any methods used to examine subgroups and interactions	Page 4
		(*c*) Explain how missing data were addressed	Page 4
		(*d*) If applicable, describe analytical methods taking account of sampling strategy	N/A
		(*e*) Describe any sensitivity analyses	N/A
**Results**			
Participants	13*	(a) Report numbers of individuals at each stage of study—eg numbers potentially eligible, examined for eligibility, confirmed eligible, included in the study, completing follow-up, and analysed	Page 4
		(b) Give reasons for non-participation at each stage	N/A
		(c) Consider use of a flow diagram	Page 4 (referred)
Descriptive data	14*	(a) Give characteristics of study participants (eg demographic, clinical, social) and information on exposures and potential confounders	Page 5
		(b) Indicate number of participants with missing data for each variable of interest	Page 6

For peer review only - http://bmjopen.bmj.com/site/about/guidelines.xhtml

Outcome data	15*	Report numbers of outcome events or summary measures	Page 6
Main results	16	(*a*) Give unadjusted estimates and, if applicable, confounder-adjusted estimates and their precision (eg, 95% confidence interval). Make clear which confounders were adjusted for and why they were included	N/A, descriptive analyses
		(*b*) Report category boundaries when continuous variables were categorized	Page 5-6
		(*c*) If relevant, consider translating estimates of relative risk into absolute risk for a meaningful time period	N/A
Other analyses	17	Report other analyses done—eg analyses of subgroups and interactions, and sensitivity analyses	N/A
**Discussion**			
Key results	18	Summarise key results with reference to study objectives	Page 10
Limitations	19	Discuss limitations of the study, taking into account sources of potential bias or imprecision. Discuss both direction and magnitude of any potential bias	Page 11
Interpretation	20	Give a cautious overall interpretation of results considering objectives, limitations, multiplicity of analyses, results from similar studies, and other relevant evidence	Page 11
Generalisability	21	Discuss the generalisability (external validity) of the study results	Page 12
**Other information**			
Funding	22	Give the source of funding and the role of the funders for the present study and, if applicable, for the original study on which the present article is based	Page 12

*Give information separately for exposed and unexposed groups.

Note: An Explanation and Elaboration article discusses each checklist item and gives methodological background and published examples of transparent reporting. The STROBE checklist is best used in conjunction with this article (freely available on the Web sites of PLoS Medicine at http://www.plosmedicine.org/, Annals of Internal Medicine at http://www.annals.org/, and Epidemiology at http://www.epidem.com/). Information on the STROBE Initiative is available at www.strobe-statement.org.

For peer review only

For peer review only - http://bmjopen.bmj.com/site/about/guidelines.xhtml

Standards for Reporting Qualitative Research (SRQR)*

http://www.equator-network.org/reporting-guidelines/srqr/

Title and abstract

	Page/line no(s).
**Title** - Concise description of the nature and topic of the study Identifying the study as qualitative or indicating the approach (e.g., ethnography, grounded theory) or data collection methods (e.g., interview, focus group) is recommended	Page 1/Line 1
**Abstract** - Summary of key elements of the study using the abstract format of the intended publication; typically includes background, purpose, methods, results, and conclusions	Page 1/Line 14-36

Introduction

**Problem formulation** - Description and significance of the problem/phenomenon studied; review of relevant theory and empirical work; problem statement	Page 2/Line 53-77
**Purpose or research question** - Purpose of the study and specific objectives or questions	Page 2/Line 80-82

Methods

**Qualitative approach and research paradigm** - Qualitative approach (e.g., ethnography, grounded theory, case study, phenomenology, narrative research) and guiding theory if appropriate; identifying the research paradigm (e.g., postpositivist, constructivist/ interpretivist) is also recommended; rationale**	Page 2/Line 98
**Researcher characteristics and reflexivity** - Researchers' characteristics that may influence the research, including personal attributes, qualifications/experience, relationship with participants, assumptions, and/or presuppositions; potential or actual interaction between researchers' characteristics and the research questions, approach, methods, results, and/or transferability	Page 3/ Line 116-119
**Context** - Setting/site and salient contextual factors; rationale**	Page 3/ Line 85-93
**Sampling strategy** - How and why research participants, documents, or events were selected; criteria for deciding when no further sampling was necessary (e.g., sampling saturation); rationale**	Page 3/ Line 114-121
**Ethical issues pertaining to human subjects** - Documentation of approval by an appropriate ethics review board and participant consent, or explanation for lack thereof; other confidentiality and data security issues	Page 3/Line 100-102
**Data collection methods** - Types of data collected; details of data collection procedures including (as appropriate) start and stop dates of data collection and analysis, iterative process, triangulation of sources/methods, and modification of procedures in response to evolving study findings; rationale**	Page 3/ Line 114-125

For peer review only

For peer review only - http://bmjopen.bmj.com/site/about/guidelines.xhtml

**Data collection instruments and technologies** - Description of instruments (e.g., interview guides, questionnaires) and devices (e.g., audio recorders) used for data collection; if/how the instrument(s) changed over the course of the study	Page 3/Line 148-150
**Units of study** - Number and relevant characteristics of participants, documents, or events included in the study; level of participation (could be reported in results)	Page 3/ Line 114-116
**Data processing** - Methods for processing data prior to and during analysis, including transcription, data entry, data management and security, verification of data integrity, data coding, and anonymization/de-identification of excerpts	Page 3/ Line 156-167
**Data analysis** - Process by which inferences, themes, etc., were identified and developed, including the researchers involved in data analysis; usually references a specific paradigm or approach; rationale**	Page 3/ Line 158-160
Techniques to enhance trustworthiness - Techniques to enhance trustworthiness and credibility of data analysis (e.g., member checking, audit trail, triangulation); rationale**	Page 3/ Line 160/163

Results/findings

**Synthesis and interpretation** - Main findings (e.g., interpretations, inferences, and themes); might include development of a theory or model, or integration with prior research or theory	Page 7/ Line 209-215
**Links to empirical data** - Evidence (e.g., quotes, field notes, text excerpts, photographs) to substantiate analytic findings	Page 8/Line 218-261

Discussion

**Integration with prior work, implications, transferability, and contribution(s) to the field** - Short summary of main findings; explanation of how findings and conclusions connect to, support, elaborate on, or challenge conclusions of earlier scholarship; discussion of scope of application/generalizability; identification of unique contribution(s) to scholarship in a discipline or field	Page 10/Line 279-319
**Limitations** - Trustworthiness and limitations of findings	Page 11/Line 353-356

Other

**Conflicts of interest** - Potential sources of influence or perceived influence on study conduct and conclusions; how these were managed	Page 12/Line 386
**Funding** - Sources of funding and other support; role of funders in data collection, interpretation, and reporting	Page 12/Line 381-383

*The authors created the SRQR by searching the literature to identify guidelines, reporting standards, and critical appraisal criteria for qualitative research; reviewing the reference lists of retrieved sources; and contacting experts to gain feedback. The SRQR aims to improve the transparency of all aspects of qualitative research by providing clear standards for reporting qualitative research.

For peer review only - http://bmjopen.bmj.com/site/about/guidelines.xhtml

**The rationale should briefly discuss the justification for choosing that theory, approach, method, or technique rather than other options available, the assumptions and limitations implicit in those choices, and how those choices influence study conclusions and transferability. As appropriate, the rationale for several items might be discussed together.

Reference:

O'Brien BC, Harris IB, Beckman TJ, Reed DA, Cook DA. **Standards for reporting qualitative research: a synthesis of recommendations.** *Academic Medicine*, Vol. 89, No. 9 / Sept 2014 DOI: 10.1097/ACM.0000000000000388

For peer review only
